# A Randomised Trial Evaluating the Safety and Immunogenicity of the Novel Single Oral Dose Typhoid Vaccine M01ZH09 in Healthy Vietnamese Children

**DOI:** 10.1371/journal.pone.0011778

**Published:** 2010-07-26

**Authors:** Tran Tinh Hien, Nguyen Thi Dung, Nguyen Thanh Truong, Ninh Thi Thanh Van, Tran Nguyen Bich Chau, Nguyen Van Minh Hoang, Tran Thi Thu Nga, Cao Thu Thuy, Pham Van Minh, Nguyen Thi Cam Binh, Tran Thi Diem Ha, Pham Van Toi, To Song Diep, James I. Campbell, Elaine Stockwell, Constance Schultsz, Cameron P. Simmons, Clare Glover, Winnie Lam, Filipe Marques, James P. May, Anthony Upton, Ronald Budhram, Gordon Dougan, Jeremy Farrar, Nguyen Van Vinh Chau, Christiane Dolecek

**Affiliations:** 1 The Hospital for Tropical Diseases, Ho Chi Minh City, Vietnam; 2 Oxford University Clinical Research Unit, Wellcome Trust Major Overseas Programme, The Hospital for Tropical Diseases, Ho Chi Minh City, Vietnam; 3 Centre for Tropical Medicine, University of Oxford, Oxford, United Kingdom; 4 Academic Medical Center, Center for Poverty-related Communicable Diseases, Amsterdam, The Netherlands; 5 London, United Kingdom; 6 Emergent Product Development UK Ltd., Wokingham, United Kingdom; 7 The Wellcome Trust Sanger Institute, Hinxton, Cambridge, United Kingdom; 8 The London School of Hygiene and Tropical Medicine, London, United Kingdom; Universidad Peruana Cayetano Heredia, Peru

## Abstract

**Background:**

The emergence of drug resistant typhoid fever is a major public health problem, especially in Asia. An oral single dose typhoid vaccine would have major advantages. M01ZH09 is a live oral single dose candidate typhoid vaccine containing *Salmonella enterica* serovar Typhi (Ty2 *aroC* ^−^ *ssaV* ^−^) ZH9 with two independently attenuating deletions. Studies in healthy adults demonstrated immunogenicity and an acceptable safety profile.

**Objectives:**

We conducted a randomised placebo controlled, single-blind trial to evaluate the safety and immunogenicity of M01ZH09 in healthy Vietnamese children aged 5 to 14 years.

**Methods:**

Subjects were randomly assigned to receive either a nominal dose of 5×10^9^ CFU of M01ZH09 or placebo and were followed up for 28 days. The primary safety outcome was the proportion of subjects with any adverse event attributed to M01ZH09. The primary immunogenicity endpoint was the proportion of subjects who showed a positive immune response to M01ZH09 in the *Salmonella* Typhi lipopolysaccharide (LPS) specific serum IgA and IgG ELISA.

**Principal Findings:**

One hundred and fifty-one children were enrolled, 101 subjects received M01ZH09 and 50 subjects received placebo. An intention to treat analysis was conducted. There were no serious adverse events and no bacteraemias. In the M01ZH09 group, 26 (26%; 95% CI, 18–5%) of 101 subjects experienced adverse events compared to 11 (22%; 95% CI, 12–36%) of 50 subjects in the placebo group (odds ratio (OR) [95%CI]  = 1.23 [0.550–2.747]; *p* = 0.691). Faecal shedding of *S.* Typhi (Ty2 *aroC* ^−^ *ssaV* ^−^) ZH9 was detected in 51 (51%; 95% CI, 41–61%) of 100 M01ZH09 subjects. No shedding was detected beyond day 3. A positive immune response, defined as 70% increase (1.7 fold change) in LPS specific serum IgG (day 14 or 28) and/or 50% increase (1.5 fold change) in LPS specific serum IgA (day 7 or 14) from baseline was detected in 98 (97%; 95% CI, 92–99%) of 101 M01ZH09 recipients and 8 (16%; 95% CI, 7–29%) of 50 placebo recipients. Twenty-eight (100%; 95% CI, 88–100%) of 28 vaccine recipients who were evaluated in the LPS specific IgA ELISPOT assay showed a positive response compared to none of the 14 placebo recipients tested.

**Conclusions:**

This was the first phase II trial of a novel oral candidate typhoid vaccine in children in an endemic country. M01ZH09 had an appropriate safety profile and was immunogenic in children.

**Trial Registration:**

Controlled-trials.comISRCTN91111837

## Introduction

Typhoid fever remains a major public health burden in developing countries with approximately 21 million cases and more than 210 000 deaths worldwide per year [1].

Drug resistant typhoid fever has emerged and spread globally narrowing the treatment options [2]–[4]. The World Health Organization therefore recommends that countries should consider the programmatic use of typhoid vaccines for controlling endemic diseases and that the immunization of school age and/or preschool age children should be undertaken particularly in areas where antibiotic resistant *Salmonella enterica* serovar Typhi (*S.* Typhi) is prevalent [5].

Two licensed and safe typhoid vaccines are available. The oral live attenuated Ty21a vaccine is moderately immunogenic and needs to be administered in three to four doses. Ty21a enteric-coated capsules and Ty21a liquid formulation (which is currently not manufactured) are licensed for children above 6 years and 2 years, respectively. The single dose injectable Vi polysaccharide vaccine is licensed for children above 2 years. The liquid formulation of Ty21a and Vi vaccine provide about 55 to 70% protection from culture confirmed typhoid fever and protection lasts for 3 to 5 years [5], [6].

From a public health perspective, a single dose oral typhoid vaccine would have major advantages [7], [8]. M01ZH09 (*S.* Typhi (Ty2 *aroC* ^−^ *ssaV* ^−^) ZH9) is a promising candidate of such a novel typhoid vaccine, it has a well-defined dual mechanism of attenuation [9] and has been safe and immunogenic in a single dose in Western [9]–[11] and Vietnamese adult volunteers (Hien TT, unpublished).

Historically, oral live vaccines often showed reduced immunogenicity in developing country populations compared to Western populations [12], therefore M01ZH09 was evaluated at an early stage of its development in children in an endemic country.

We describe here the results of a randomised placebo controlled trial that evaluated the safety and immunogenicity of M01ZH09 in 151 healthy Vietnamese children aged between 5 and 14 years.

## Methods

The protocol for this trial and supporting CONSORT checklist are available as supporting information, see Protocol S1 and Checklist S1.

### Study design and objectives

The study was designed as a randomised placebo controlled single blind trial to evaluate the safety and immunogenicity of the novel oral single dose live typhoid vaccine M01ZH09 in Vietnamese children aged 5 to 14 years (inclusive).

### The study site and ethical approval

The trial was conducted at the Hospital for Tropical Diseases in Ho Chi Minh City, Vietnam. Ethics approval for the trial and all trial related documents was obtained by the Oxford Tropical Research Ethics Committee (OXTREC) and by the Institutional Review Board of the Ministry of Health, Hanoi, Vietnam. The trial was conducted in accordance with the Declaration of Helsinki and its amendments and according to Good Clinical Practice guidelines and was monitored by Matrix Contract Research Ltd., UK (now Novella Clinical). The trial was also conducted under an US Investigational New Drug (IND) license.

### Participants

Healthy Vietnamese children aged 5 to 14 years (inclusive) were invited to participate in the trial. Recruitment was carried out by word of mouth and flyers. Families who were interested in the trial were invited to attend one of several information evenings at the Hospital for Tropical Diseases. At these meetings the study was presented by the principal investigator (TTH) and all questions could be discussed and answered. Families who remained interested in the trial were invited to attend the screening visit. Children were eligible if they were available during the trial period and at least one of their parents gave written informed consent for their child to participate after the trial procedures and potential risks were carefully explained by the study investigators. All children were invited to give their assent to the study and written informed assent was obtained from subjects starting at the age of 6 years. After informed consent was obtained, screening tests were performed. Children were screened by history, physical examination (including height, weight and vital signs), blood tests (biochemistry, haematology and HIV test), urine dipsticks and pregnancy tests (for female subjects of 11 years and above). Stool cultures were performed to check for the presence of *Salmonella* species.

Subjects with a history of typhoid fever, Ty21a vaccination in the last 10 years or any other typhoid vaccine in the last 5 years, any clinically significant illness, abnormal blood test results, immune suppression, positive HIV or pregnancy test were excluded. Also excluded were subjects whose body weight was under 17 kg in the 5 to 10 year old group or under 27 kg in the 11 to 14 year old group and subjects who suffered from an acute febrile illness at the time of dosing (the complete list of exclusion criteria is available in the trial protocol). Only one child per family was allowed to participate in the trial.

The results of the screening tests were reviewed and subjects who continued to meet the inclusion criteria were invited to continue in the trial.

### The M01ZH09 vaccine and dose

*S.* Typhi (Ty2 *aroC* ^−^ *ssaV* ^−^) ZH9 was constructed with a rational attenuation strategy. Two defined independently attenuating deletion mutations were introduced into *S.* Typhi Ty2. Deletion of *aroC*, encoding chorismate synthase, prevents the biosynthesis of aromatic amino acids and deprives the live vaccine bacterium of essential nutrients. Deletion of *ssaV*, encoding a structural component of the *Salmonella* pathogenicity island-2 (SPI-2) type III secretion system, prevents systemic spread of *S.* Typhi [9]. The vaccine was manufactured according to Good Manufacturing Practice protocols by Eurogentec S.A. and SynCo Bio Partners B.V; batch number M-STZH9-F16 was shipped to Vietnam. The vaccine kits were stored at 2–8°C

Previous studies in adult volunteers demonstrated that a nominal dose of 5×10^9^ CFU of the vaccine strain was immunogenic and safe [9]–[11]. The Ty21a oral typhoid vaccine capsules are licensed for adults and children above 6 years using the same dose and immunization schedule and large Ty21a field trials in children used the same dose and regimen as in adults [13]. It was therefore determined that the appropriate dose for the children's study was a nominal dose of 5×10^9^ CFU of *S.* Typhi (Ty2 *aroC* ^−^ *ssaV* ^−^) ZH9.

The vaccine (containing 5×10^9^ CFU of vaccine strain plus excipients) and the placebo (vaccine excipients only) were supplied as freeze-dried formulations in single dose vials, which were labeled identically, containing “M01ZH09 oral typhoid vaccine or placebo” but with a unique subject number corresponding to the randomisation list. The bicarbonate solution was prepared by dissolving one effervescent bicarbonate tablet (provided in the vaccine kit and containing 2.6 g sodium bicarbonate, 1.65 g ascorbic acid and 30 mg aspartame) in 150 ml of bottled drinking water (final concentration: 1.75% wt/vol sodium bicarbonate, 1.1% wt/vol ascorbic acid, and 0.02% wt/vol aspartame). The lyophilised vaccine or placebo was reconstituted in either 150 ml (for children above 10 years) or in 75 ml of the bicarbonate solution (the other 75 ml were discarded) for children below 10 years and was administered immediately.

The study used two age group specific randomisation lists, one for the 11 to 14 year old and one for the 5 to 10 year old children to ensure at least 70% children were between 5 to 10 years old.

### Intervention

On the day of vaccination (day 0) which took place within 28 days of the screening, inclusion and exclusion (including history of antibiotic medication in the last 2 weeks) criteria were reviewed. Pregnancy tests (female subjects of 11 years and above only), urine dipstick test and stool cultures were performed. Blood samples for haematology, biochemistry, ELISA and ELISPOT assays (only in children 11 years and above) were obtained. After the subjects had fasted for at least 2 hours (with the exception of drinking water), the candidate typhoid vaccine or placebo was administered.

Subjects were allocated the next age-group specific subject number and the medication pack bearing the same number was prepared and issued by the pharmacist, who was otherwise not involved in the trial. The subjects were randomly assigned to receive either M01ZH09, consisting of 5×10^9^ CFU of *S.* Typhi (Ty2 *aroC* ^−^ *ssaV* ^−^) ZH9 or the placebo reconstituted in bicarbonate solution as described above.

Volunteers were observed for at least 90 minutes at the hospital. During this time pulse and blood pressure were recorded periodically and only drinking water was provided.

Diary cards were issued for all the volunteers and all subjects received a basic hygiene kit containing soap, gloves and spatulas for the collection of stool samples. The subjects and their parents were instructed to measure and record the oral temperature of the children twice daily (morning and evening) and to record any adverse events (including headache, fever, nausea, vomiting, abdominal pain, frequency and consistency of stools and any other symptoms) for 14 days.

### Follow up procedures and monitoring of adverse events

Children were followed up daily from days 1 to 14 and again on day 28 after dosing. At these appointments diary cards were checked and adverse events and concomitant medication reviewed. A history of the last 24 hours with special emphasis on temperatures of 38.5°C and above and adverse events (diarrhoea, loss of appetite, vomiting, headache and chills) was obtained. Oral temperatures and vital signs were recorded and children were examined for signs of splenomegaly. Stool cultures were performed daily from day 1 to day 14. Blood samples for biochemistry and haematology were obtained on days 7, 14 and 28; for the LPS specific serum IgA ELISA on days 7 and 14; for the IgG ELISA on days 14 and 28 and for the LPS specific IgA antibody secreting cell (ASC) ELISPOT assay (only in subjects aged 11 years and above) on day 7. The total amount of blood taken during this study was approximately 28 ml from the 5 to 10 years old and 44 ml from the 11 to 14 years old children.

### Unscheduled visits

Subjects and parents were instructed to make additional visits to the clinic, if the child felt unwell and/or had a fever of ≥38.5°C. At these visits the subject was assessed and samples taken for culture as clinically indicated. Blood cultures to investigate for the presence of *S.* Typhi in blood would be obtained if a fever of ≥39.0°C was recorded twice over a 48 hours period, or a severe fever of ≥39.5°C was recorded once.

### Definition and reporting of serious adverse events and definition of stopping rules

There was no Data Safety and Monitoring Committee for this trial. Data from all children were reviewed daily and there were *a priori* defined stopping rules which would trigger a suspension of the trial and a safety review (Protocol S1). Serious adverse events were reported to AKOS Ltd (Hitchin, UK), a pharmacovigilance company within 24 hours.

### Detection of *Salmonella* in stool samples at the screening visit and day 0

The detection of *Salmonella* species at the screening visit and on day 0 was performed according to microbiological standard procedures. In brief, stool samples were inoculated onto MacConkey agar and xylose lysine deoxycholate (XLD) agar plates, and in 10 ml of selenite F broth. Plates and broth were incubated at 37°C overnight and the broth was sub-cultured on MacConkey and XLD agar plates the next morning. Isolates were screened using standard biochemical tests and *Salmonella* were identified by slide agglutination with specific antisera (Oxoid Ltd., UK) and API20E profiling (bioMérieux, UK).

### Detection of *S.* Typhi in stool samples

Stool samples were collected daily between days 1 and 14. Stool samples were cultured directly on deoxycholate citrate agar (DCA) Hynes plates (direct method) and in selenite F broth (enriched method), both of which were supplemented with aromatic compounds (DCA-aro and selenite F-aro, respectively) to detect *S.* Typhi, including the auxotrophic vaccine strain, in stools. Following overnight incubation at 37°C, an aliquot of the inoculated selenite F-aro broth was sub-cultured on DCA-aro Hynes plates. Suspected *S.* Typhi colonies were inoculated on brain heart infusion agar plates supplemented with aromatic compounds (BHI-aro). Oxidase negative colonies were evaluated by agglutination with Hd, Vi and O9 anti-sera (Oxoid Ltd., UK) and API20E profiling (bioMérieux, UK). Stool samples containing isolates that were positive in at least 2 out of 3 agglutinations and identified as *S.* Typhi by API20E profiling were considered to be positive for *S.* Typhi. All isolates were stored in 10% (v/v) glycerol at −80°C.

### Detection of *S.* Typhi in blood samples

Blood samples were collected into either Bactec Peds Plus/F culture bottles (1–3 ml blood; BD, USA) or Bactec Plus Aerobic/F culture bottles (4–10 ml blood; BD, USA) and supplemented with aromatic compounds. Blood cultures were incubated at 35°C in the Bactec detection system and monitored for up to 5 days. Gram stain was performed on all bottles triggering a positive reaction. Positive cultures and all cultures that were negative after 5 days of incubation were sub-cultured on XLD agar plates. Suspected *S.* Typhi colonies were sub-cultured onto BHI-aro agar plates. Oxidase negative isolates were evaluated by agglutination and API20E profiling (bioMérieux, UK) as above.

### PCR identification of *S.* Typhi isolates

Genomic DNA was isolated from glycerol stocks of *S.* Typhi isolates using a DNeasy blood and tissue kit (Qiagen, UK). Multiplex PCRs were performed using a Taq PCR core kit (Qiagen, UK). Each reaction mixture contained 200 µM dNTPs, 0.4 µM ssaV4 (5′ ATCCCCACGACTTCAGCAAG 3′) and ssaV7 (5′ CTTTCTGGCTCATCATGAGG 3′), and 0.1 µM aroC.Z1 (5′ GACAACTCTTTCGCGTAACC 3′) and aroC.Z3 (5′ TTACATCCGCATTCTGTGCC 3′), 10 ng genomic DNA and 1.25 u *Taq* DNA polymerase in a total volume of 50 µl reaction buffer. PCRs were performed for 25 cycles as follows: 94°C for 30 sec, 57°C for 30 sec and 72°C for 2.5 min. The PCR products were visualised by ethidium bromide staining and UV transillumination after electrophoresis on a 0.8% (w/v) TAE agarose gel. The expected sizes of the PCR products were 1.04 kb (*aroC*) and 2.59 kb (*ssaV*) for *S.* Typhi wild-type strains and 0.45 kb (*aroC*) and 0.70 kb (*ssaV*) for *S.* Typhi (Ty2 *aroC* ^−^ *ssaV* ^−^) ZH9.

### Detection of antibody secreting cells producing *S.* Typhi LPS specific IgA antibodies by ELISPOT assay

ELISPOT assays to detect antibody secreting cells (ACS) producing *S.* Typhi LPS specific IgA antibodies were performed on days 0 and 7 as described previously [10], [14]. In brief, whole blood was collected in heparinised cell preparation tubes (Vacutainer CPT; BD, UK) and centrifuged. Peripheral blood mononuclear cells (PBMCs) were washed, resuspended in culture medium and adjusted to three cell concentrations (1×10^7^/ml, 5×10^6^/ml and 2.5×10^6^/ml). One hundred microlitres of each concentration were added to LPS coated and uncoated wells (for subtraction of non-specific results) of nitrocellulose microtiter plates (Millipore, USA) and incubated overnight at 37°C in a 5% CO_2_ incubator. PBMC collected from a healthy volunteer who had received three doses of Ty21a (Vivotif, Berna, Switzerland) were included as positive control and PBMC from a non-vaccinated person as negative control. Plates were washed and an alkaline phosphatase-conjugated anti-human IgA antibody (Immune Systems Ltd., UK) was added and incubated for one hour. Plates were washed and spots were visualised by the addition of 5-bromo-4-chloro-3-indolyl-phosphate/nitro blue tetrazolium (BCIP/NBT) substrate. Antibody secreting cell (ASC) spots were counted manually using an inverted microscope. If more than 100 spots per well were present, the result was described as “too many spots to be counted”. A positive response in the ELISPOT assay on day 7 was defined as ≥4 IgA ASC specific for LPS per 10^6^ PBMCs and a negative response as <4 IgA ASC specific for LPS per 10^6^ PBMCs. Subjects with a day 0 result of ≥4 ASC per 10^6^ PBMC were excluded from the ELISPOT analysis. ELISPOT assays were performed at the Hospital for Tropical Diseases.

### Analysis of *S.* Typhi LPS specific serum IgG and IgA by ELISA and definition of a positive immune response

Quantitative ELISA methods for measuring *S.* Typhi LPS specific serum IgG and IgA were developed and qualified by Emergent Product Development UK Ltd using serum samples from recipients of M01ZH09 who participated in prior clinical trials and who had given informed consent for retention and usage of their samples. For the IgG ELISA, serum which demonstrated more than 4-fold increase from pre-dose in a previously described end point titre assay [11] were pooled and used as reference standard. The LPS specific IgG concentration in the standard serum was set arbitrarily at 30000 units/ml. For the IgA ELISA, serum from past recipients of M01ZH09 who demonstrated positive response in IgA ELISPOT were pooled and used as reference standard. The LPS specific IgA concentration in the standard serum was set arbitrarily at 100 units/ml.

Precision was evaluated as part of the assay qualification exercise. Variance component analysis was carried out (PRISM Training & Consultancy Ltd, UK) to calculate the standard error of measured sample means and the least significant difference (LSD) between two samples at the 1% significance level; this was used as the cut-off value for a positive result in the respective assay. A positive serum IgG response was defined as a 70% increase (fold change of 1.7) as compared to the corresponding baseline sample, whereas a positive serum IgA response was defined as a 50% increase (fold change of 1.5) as compared to baseline.

Serum samples for measurement were frozen at −20°C and shipped to Emergent Product Development UK Ltd for the ELISA analyses. For the IgG ELISA, microtiter plates were coated with *S.* Typhi LPS, washed and then blocked. Washing occurred between each step. Calibration standards and diluted test samples were added, and the plates were incubated. Bound IgG was detected using an anti-human IgG antibody conjugated to horseradish peroxidise (HRP) (Dako, Denmark) followed by the addition of 3,3′,5,5′-Tetramethylbenzidine (TMB) substrate. The plates were read at 450 nm within 30 minutes of stopping the reactions with 0.3 mol/L sulphuric acid. The standard curve was constructed by plotting the optical densities (ODs) of standards against concentrations and fitted by a 4-parameter logistic equation (SoftMax® Pro 4.6, Molecular Devices, USA). The concentration of LPS specific IgG in each sample was determined from the standard curve. The IgA quantitative ELISA was performed in a similar manner, except using a double detection system of biotinylated anti-human IgA antibody (Southern Biotech, USA) followed by streptavidin-HRP conjugate (Dako, Denmark).

### Outcomes of the study

#### Safety Outcomes

The primary safety endpoint was the proportion of subjects with any adverse events attributed to M01ZH09. The secondary safety endpoint was the proportion of subjects with any serious related adverse events; any related or unrelated adverse events; persisting faecal shedding of *S.* Typhi (Ty2 *aroC* ^−^ *ssaV* ^−^) ZH9 after day 7; and/or had a fever of 38.5°C or greater in the 14 days post vaccination, withdrew from the trial due to adverse events, including bacteraemia, and/or had clinically significant changes in laboratory parameters related to the candidate vaccine.

All subjects who received a dose of the vaccine or placebo were analysed in the safety population. Post-vaccination adverse events were categorised according to body system and preferred term using the Medical Dictionary for Regulatory Activities (MedDRA, Version 9.1), allocated before unblinding. Adverse events were graded by severity (mild, moderate, severe) and judged for the relatedness to the study vaccine (unlikely, possibly, probably) by the investigator. Only possibly and probably related adverse events were attributed to the vaccine. Moderate fever was defined as an oral temperature of ≥38.5°C and severe fever as an oral temperature of ≥39.5°C. Moderate diarrhoea was defined as more than 4 unformed stools and severe diarrhoea as more than 6 unformed stools in a 24 hour period or evidence of significant dehydration. All adverse events were recorded in the CRFs and monitored until return to normal.

The numbers and proportion of subjects reporting adverse events were listed by body system. A per subject analysis of adverse events was performed e.g., if a subject reported the same adverse event on three occasions that adverse event was only counted once. Subjects reporting more than one adverse event per body system were counted only once in that body system total.

#### Immunogenicity Outcomes

The primary immunogenicity endpoint was the proportion of subjects who developed a positive immune response to *S.* Typhi LPS defined by an increase of 70% (1.7 fold change) in LPS specific serum IgG on day 14 or 28 and/or an increase of 50% (1.5 fold change) in LPS specific serum IgA on day 7 or 14 compared to baseline.

The secondary immunogenicity endpoints were defined as the proportion of subjects who developed a positive immune response in each of the following assessments: *S.* Typhi LPS specific IgA ELISA assay on days 7 or 14, *S.* Typhi LPS specific IgG ELISA assay on days 14 or 28 and *S.* Typhi LPS specific IgA ELISPOT on day 7. A positive ELISPOT was defined as ≥4 IgA antibody secreting cells specific for *S.* Typhi LPS per 10^6^ PBMCs.

### Sample Size

The planned sample size was 150 subjects, of whom at least 70% should be aged 10 years or younger, as this was the target age of the vaccine, randomised to M01ZH09 or placebo in a 2∶1 ratio.

No formal sample size calculation was considered appropriate; it was aimed to include a sufficient sample size to assess safety and immunogenicity based on previous observations in adult studies and immunogenicity rates of licensed typhoid vaccines in children.

### Randomisation procedures and assignment of intervention (sequence generation, allocation concealment, implementation)

Randomisation codes were computer generated in blocks of 9 by Statwood Ltd, UK. The vaccine and the placebo were labeled identically but with a unique subject number corresponding to the randomisation list. The study used two age group specific randomisation lists, one for the 11 to 14 year old and one for the 5 to 10 year old children to ensure at least 70% children were between 5 to 10 years old.

Subjects were allocated the next age-group specific subject number in strict numerical sequence from this list and the medication pack bearing the same number was prepared by the pharmacist.

### Blinding

This study was formally a single blind study due to slight differences in taste and aroma between the treatment preparations but it was conducted under the principles of a double blind study. M01ZH09 and placebo were packaged and labeled identically but with a unique sequential number. Possible sources of unblinding could have been the preparation of the vaccine, therefore the study pharmacist was otherwise not involved in the trial. The subjects were asked to not report the taste of the vaccine. Microbiology results were not reviewed by the investigators for at least 14 days after vaccination to avoid potential unblinding through shedding in stools. Immunology results were not reviewed by the investigators. The study site received code break envelopes in case an emergency made unblinding for a single subject necessary. No codes were broken during this study. The unblinding of treatment allocations took place after the trial had been completed and the whole database had been entered and locked.

### Data collection, data entry and statistical methods

All data were recorded in Case Record Forms (CRFs). CRFs were reviewed and collected by the study monitor. Data entry, data management and statistical analysis were conducted by Statwood, UK using SAS® software (version 9.1). Data were double entered and analysed according to an *a priori* defined statistical analysis plan which included the definition of all subject populations and the trial endpoints. The safety population included all subjects who received the study medication. The intention to treat (ITT) population comprised all dosed subjects who had any post-dose immunogenicity data available. The per protocol (PP) population excluded major protocol violators (failure to meet the inclusion/exclusion criteria, to comply with the study medication or use of other vaccinations or antibiotics two weeks before until 2 weeks after vaccination, or use of antacids or proton-pump inhibitors prior to vaccination and/or did not provide samples for the ELISAs). The protocol stated that a confirmatory analysis of the primary immunogenicity endpoint in the PP population was planned if more than 5% of subjects were excluded.

The proportion of subjects who experienced post-dose adverse events was presented together with their two-sided 95% confidence intervals (95%CI). Post dose adverse events, adverse events considered to be related to the vaccine and adverse events that occurred in more than 10% of the trial population were tested using a two-sided Fisher's exact test to compare between the two groups.

The proportion of subjects who developed a positive immune response was presented together with their two-sided 95% confidence intervals calculated by using an exact Binomial distribution. The treatment difference and associated 95% confidence interval were presented as above. All available data from withdrawn subjects was included in the analysis.

## Results

### Participant flow and recruitment

**Figure 1:**
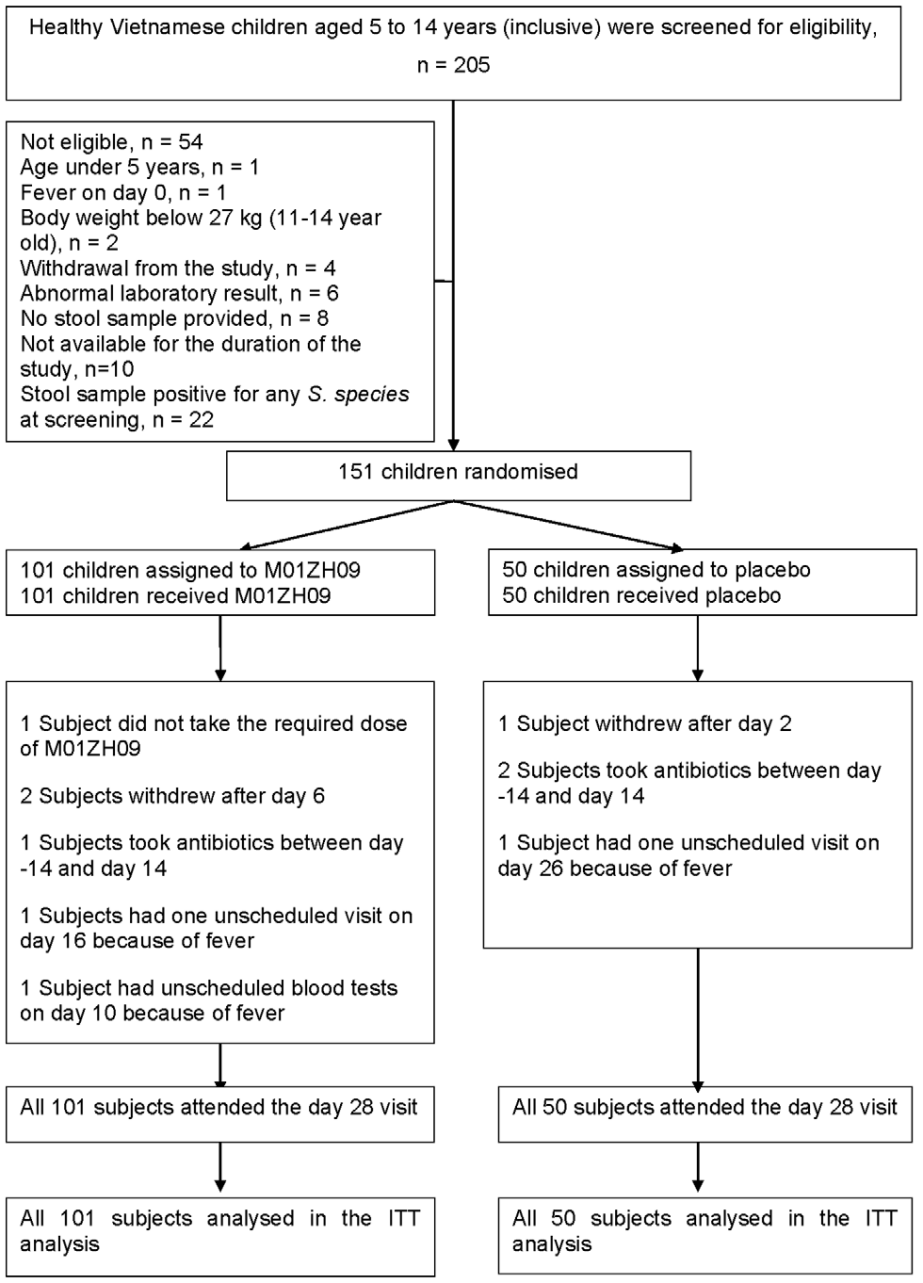
Flow of subjects.

The trial was conducted between April and July 2007. In total, 205 healthy Vietnamese children between 5 and 14 years (inclusive) were screened for eligibility. Fifty-four children were not eligible (Figure 1), the most common reasons were unavailability for the whole study period (n = 10) and a positive stool culture for *Salmonella* species at screening (non-typhoid *Salmonella*, n = 22). No *S.* Typhi or *S.* Paratyphi A were detected in stools at the screening visits.

One hundred and fifty-one children were randomised, 101 children received the candidate typhoid vaccine M01ZH09 and 50 children received placebo. All subjects fulfilled the inclusion and exclusion criteria at screening and dosing, however two subjects (both in the M01ZH09 group) had clinically significant elevated white blood counts (16.3 and 18.2×10^9^/L respectively) on day 0, these results were only available after dosing.

One subject in the M01ZH09 group vomited after taking approximately 50% of the required vaccine dose. The subject agreed to take another dose, but failed to retain it. Three subjects withdrew from the study, one placebo recipient withdrew due to non-compliance (refused to provide stool samples) after day 2 and two vaccine recipients left the study after day 6 (one wished to withdraw, the second subject went on holiday). All three subjects attended the day 28 visit. The remaining subjects attended all study visits. Two subjects had unscheduled visits. One subject in the vaccine group attended the clinic on day 16 because of fever of 38.1°C and one subject in the placebo group returned on day 26 with a temperature of 38.0°C. Blood cultures were obtained from both subjects and both cultures were negative. One M01ZH09 recipient had unscheduled tests performed. The subject presented on day 10 with a temperature of 38.6°C and reported diarrhoea, vomiting and fever on the previous day. The white blood count was elevated with 14.2×10^9^/L and the blood culture result was negative.

### Numbers analysed

All 151 children who were randomised and received either M01ZH09 (n = 101) or placebo (n = 50) constituted the intention to treat (ITT) population. Seven subjects, 4 in the vaccine group and 3 in the placebo group were protocol violators (see Fig. 1) and were excluded from the per protocol (PP) population. The analysis of the primary endpoints in the PP population was planned if more than 5% of subjects were excluded from the ITT. The PP population comprised 95% (144/151) of subjects and therefore no per protocol analysis was conducted.

All outcomes were evaluated for the ITT population.

### Baseline data

The two groups did not differ significantly at enrolment in terms of sex, age and laboratory parameters (Table 1). One hundred and seven (71%) children were aged 10 years or younger.

**Table 1:** Baseline characteristics of the subjects on day 0 (Intention to Treat population).

Characteristics	M01ZH09 group (n = 101)	Placebo group (n = 50)	Overall (n = 151)
Age in years	9 (5−14)	9 (5−14)	9 (5−14)
Number of males (%)	54 (53)	27 (54)	81 (54)
Weight in kilograms	28 (17−53)	26.5 (17−66)	27 (17−66)
Height in cm	132 (97−165)	130.50 (100−165)	132 (97−165)
Oral temperature in °C	36.80 (36.1−37.5)	36.75 (35.1−37.4)	36.80 (35.1−37.5)
Haemoglobin, g/dl*	13.3 (10.5−15.3)	13.4 (10.9−15.5)	13.3 (10.5−15.5)
White cell count, 10^9^/L∧	7.2 (5−18.2)	8.0 (4.9−10.7)	7.4 (4.9−18.2)
Lymphocytes, % ∧	39.6 (14.9−55.5)	39.9 (21.4−67.4)	39.7 (14.9−67.4)
Neutrophils, % ∧	48.6 (29.2−70)	50.5 (19.5−73.8)	49.9 (19.5−73.8)
Monocytes, % ∧	5.3 (2.3−12.3)	5.0 (1.4−13.2)	5.2 (1.4−13.2)
Basophiles, % ∧	0.3 (0−0.7)	0.3 (0−0.7)	0.3 (0−0.7)
Eosinophiles, % ∧	4.0 (0.1−18.4)	3.0 (0.4−15.2)	3.8 (0.1−18.4)
Platelet count, 10^9^/L^&^	295 (190−503)	311 (190−467)	300 (190−503)
Serum Aspartate Aminotransferase AST, U/L	27 (14−52)	26 (14−51)	27 (14−52)
Serum Alanine Aminotransferase ALT, U/L	16 (6−58)	17 (5−43)	16 (5−58)
Creatinine, mM/L	0.47 (0.33−0.70)	0.47 (0.27−0.73)	0.47 (0.27−0.73)
Stool culture positive for *Salmonella species*	7	5	12

All data are presented as median (range) unless otherwise specified.

Data from one*, two∧ and seven^&^ subjects not available.

Two subjects in the M01ZH09 group had clinically significant elevated white blood counts on day 0 (see above). Twelve subjects in total, five in the placebo group and seven in the M01ZH09 group (this included the subject who vomited on the day of dosing) had a positive stool culture for non-typhoid *Salmonella* on day 0, these results were only available after dosing.

### Protocol deviations and modifications

The protocol stated that the primary safety endpoint would be the proportion of subjects reporting serious adverse events attributed to M01ZH09. Due to concerns that these numbers might be small and would not be sufficient to detect a difference in safety between the two groups, the primary endpoint was changed to the proportion of subjects with any related adverse event. This change was made after the completion of the clinical phase but prior to database lock and unblinding.

### Outcomes and estimation

#### Safety Outcomes

There were no serious adverse events, no bacteraemia and none of the subjects withdrew due to adverse events in this trial.

Similar proportion of subjects reported adverse events in both treatment groups during the 28 days of follow up, but the total number of events was higher in the M01ZH09 group. In the vaccine group, 26 (26%; 95%CI, 18–35%) of 101 subjects reported 64 adverse events compared to 11 (22%; 95%CI, 12–36%) of 50 subjects in the placebo group who reported 17 adverse events (odds ratio (OR) [95%CI]  = 1.23 [0.550−2.747]; *p* = 0.691) (Table 2). Repeated occurrences of a particular adverse event in the same subject were included in the total number of 64 and 17 adverse events**,** respectively. Of the 64 adverse events reported by M01ZH09 recipients, 55 were mild (8 of these were considered to be related to the candidate vaccine), 8 moderate (one related) and one was severe and related to M01ZH09 (Table 2). Of the 17 adverse events in the placebo group, 12 were mild, 5 moderate and none was related.

**Table 2:** Incidence of adverse events after vaccination during 28 days of follow up (Intention to Treat population).

Event	M01ZH09 (n = 101)		Placebo (n = 50)	
	Number of subjects	Percent (95% CI)	Number of subjects	Percent (95% CI)
**Any adverse event**	**26**	**26 (18−35)**	**11**	**22 (12−36)**
**Gastrointestinal disorders (%)**	**12**	**12 (6−20)**	**1**	**2 (0−11)**
* related to vaccine*	3	3	0	0
Abdominal pain (%)	8	8	0	0
* related to vaccine*	2	2	0	0
Constipation (%)	2	2	0	0
Diarrhoea	5	5	1	2
* related to vaccine*	3	3	0	0
Nausea	3	3	0	0
* related to vaccine*	1	1	0	0
Vomiting	3	3	0	0
**General disorders and administration site conditions**	**9**	**9 (4−16)**	**5**	**10 (3−22)**
Chills	1	1	1	2
Fatigue	0	0	2	4
Pyrexia	8	8	3	6
* related to vaccine and severe* *	1	1	0	0
**Infections and infestations**	**1**	**1 (0−5)**	**0**	**0 (0−7)**
Viral infection	1	1	0	0
**Investigations**	**4**	**4 (1−10)**	**0**	**0 (0−7)**
Urine colour abnormal	2	2	0	0
White blood cell count increased	2	2	0	0
**Metabolism and nutrition disorders**	**3**	**3 (1−8)**	**0**	**0 (0−7)**
Anorexia	1	1	0	0
* related to vaccine*	1	1	0	0
Decreased appetite	2	2	0	0
**Nervous system disorders**	**9**	**9 (4−16)**	**1**	**2 (0−11)**
Headache	9	9	1	2
* related to vaccine*	2	2	0	0
**Respiratory, thoracic and mediastinal disorders**	**6**	**6 (2−12)**	**7**	**14 (6−27)**
Cough	6	6	7	14
Rhinorrhoea	1	1	0	0
**Skin and subcutaneous tissue disorders**	**1**	**1 (0−5)**	**1**	**2 (0−11)**
Rash	1	1	1	2
**Vascular disorders**	**1**	**1 (0−5)**	**0**	**0 (0−7)**
Hypertension	1	1	0	0

Per-subject analysis of adverse events (unlikely, possibly and probably related to the vaccine) reported during 28 days of follow up. Subjects could experience more than one adverse event. Each adverse event was only counted once for each subject and system class. There were 56 adverse events in the M01ZH09 group and 16 in the placebo group, when repeated occurrences of a particular event in the same patient were only counted once. Adverse events that were possibly or probably related to the vaccine are presented in italic.

*One severe adverse event was reported.

Four (4%) M01ZH09 recipients experienced 10 adverse events that were related to the candidate vaccine compared to none in the placebo group (*p* = 0.302). Of these, 8 were mild, one moderate (diarrhoea) and one event of pyrexia was severe. The moderate and the severe related adverse events occurred in the same subject. This subject had a normal temperature on day 0, but the pre-dose blood test showed an elevated white blood cell count (16.3×10^9^/L). The subject experienced five post vaccination adverse events occurring on day 0, including fever of 38.5 and 39.0°C, diarrhoea, headache, abdominal pain and anorexia. The subject received paracetamol and recovered.

Similar proportions of subjects experienced fever post vaccination (Table 2), only one subject reported fever related to M01ZH09 (see above).

Adverse events classified as gastrointestinal disorders, nervous system disorders and investigations were experienced by a higher proportion of M01ZH09 recipients (Table 2). Twelve (12%) vaccine recipients experienced gastrointestinal disorders compared to 1 (2%) placebo recipient (*p* = 0.061). Nervous system disorders (headache) occurred in 9 (9%) vaccine recipients compared to 1 (2%) placebo recipient (*p* = 0.166) and investigations were reported by 4 (4%) vaccine recipients compared to none of the placebo recipients (*p* = 0.302).

Cough was the most frequently reported adverse event, occurring in 6 (6%) M01ZH09 recipients versus 7 (14%) placebo recipients (*p* = 0.124).

On day 1 after vaccination, faecal shedding of *S.* Typhi occurred in 47 (49%) of 95 vaccine recipients; shedding was detected by the direct method in 11 (12%) subjects and by the enriched method of culturing stools in 36 (38%) subjects. On day 2 after vaccination, faecal shedding was detected in 12 (12%) of 97 subjects (in 1 (1%) subject by direct and in 11 (11%) subjects by enriched method). Only one (1%) of 98 subjects experienced shedding on Day 3 (detected by enrichment method). In total, 51 (51%; 95% CI, 41–61%) of 100 M01ZH09 subjects experienced shedding on either days 1, 2 or 3 and no subjects experienced shedding on day 4 after vaccination or later.

The presence of *S.* Typhi was detected in the stools of 1 (2%; 95%CI, 0–11%) of 50 subjects in the placebo group. This occurred on day 2 and was detected using the enriched method. No fever or adverse events were recorded for this subject. The finding of a positive stool culture for *S.* Typhi in a placebo subject was only available after unblinding of the trial. All previous and all sequential stool cultures up to day 14 of this subject were negative. This isolate was identified as the vaccine strain *S.* Typhi (Ty2 *aroC* ^−^ *ssaV* ^−^) ZH9 by subsequent PCR analysis.

Seven (7%) of 101 M01ZH09 recipients and 3 (6%) of 50 placebo recipients were detected to have a positive stool culture for non-typhoid *Salmonella* between day 1 and 14 after vaccination.

#### Immunogenicity Outcomes

Ninety-eight (97%; 95%CI, 92–99%) of 101 subjects in the M01ZH09 group and 8 (16.0%; 95%CI, 7–29%) of 50 subjects in the placebo group developed a positive immune response in either the *S.* Typhi LPS specific serum IgG or IgA ELISA, defined as the primary endpoint (Table 3). The difference in proportions of responders between the vaccine group and the placebo group was 81.0% (95% CI; 68–89%), the lower limit of the 95% CI of this difference was greater than 50% and fulfilled the *a priori* defined criterion for an acceptable immune response.

**Table 3:** Proportions of responders to the candidate typhoid vaccine M01ZH09 (Intention to Treat population).

	M01ZH09 group n = 101			Placebo group n = 50		
	No.	Positive Immune response, no. (%)	95% CI	No.	Positive Immune response, no. (%)	95% CI
**Detected in IgA ELISA assay**						
**Day 7**	99	88 (89)	81–94	49	1 (2)	0–11
**Day 14**	99	92 (93)	86–97	49	1 (2)	0–11
**Day 7 or day 14**	99	94 (95)	89–98	49	2 (4)	1–14
**Detected in IgG ELISA assay**						
**Day 14**	99	91 (92)	85–97	49	6 (12)	5–25
**Day 28**	101	90 (89)	81–94	50	6 (12)	5–24
**Day 14 or 28**	101	93 (92)	85–97	50	8 (16)	7–29
**Detected in either IgA or IgG ELISA assay**						
**Day 7, 14 or 28**	101	98 (97)	92–99	50	8 (16)	7–29
**Detected in IgA ELISPOT** *						
**Day 7**	28	28 (100)	88–100	14	0 (0)	0–23

No., number of subjects who provided samples.

A positive immune response in the ELISA assay was defined by an increase of 50% (1.5 fold change) in LPS specific serum IgA and/or an increase of 70% (1.7 fold change) in LPS specific serum IgG compared to baseline.

*44 subjects aged 11 years and above (29 subjects in the M01ZH09 group and 15 subjects in the placebo group) were eligible for the ELISPOT.

A positive ELISPOT result was defined as ≥4 IgA antibody secreting cells specific for *S.* Typhi LPS per 10^6^ PBMCs. None of the subjects had a positive day 0 ELISPOT result.

**Figure 2:**
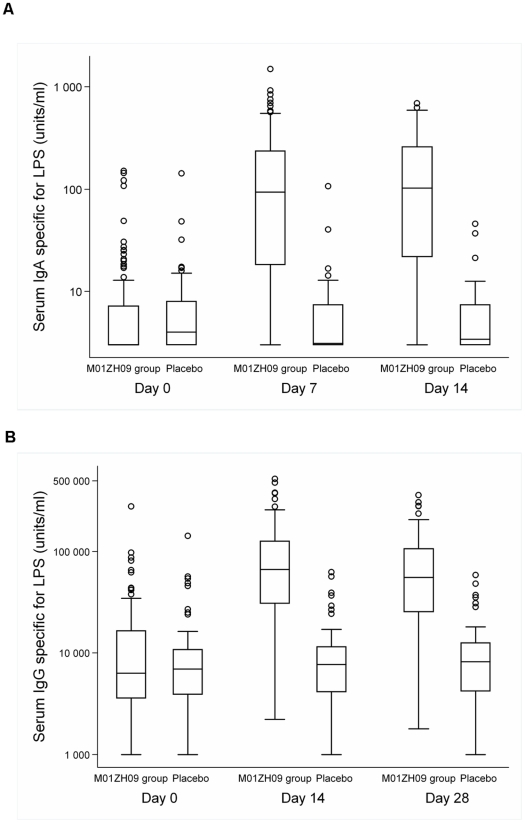
Time course of LPS specific serum IgA (A) and IgG (B) antibody levels according to vaccination groups (Intention to Treat population). Box and whisker plots showing the distribution of antibodies according to time point and vaccination groups. The horizontal line within each box represents the median, the top and bottom of each box represents the 75^th^ and 25^th^ percentiles, respectively, and the Ibar represents the highest and lowest values within 1.5 times the interquartile range. Circles show outliers.

Median baseline LPS specific antibody levels were comparable in both groups (Figure 2). In the M01ZH09 group, median IgA antibody levels increased from 3 (IQR; 3–7.2) units/ml at baseline to 94 (IQR; 19.8–231.5) units/ml and 103 (IQR; 23.9–253.5) units/ml on days 7 and 14 respectively. On day 7, the 88 immune responders in the vaccine group (Table 3) displayed a median 16.4 (IQR 3.75–60.25) fold rise in serum IgA antibodies relative to baseline (Table 4, Supplements).

**Table 4:** Serum IgA and IgG antibody levels specific for *S*. Typhi LPS (Intention to Treat population).

	M01ZH09 group n = 101		Placebo group n = 50	
Day	Median units/ml	IQR	Median units/ml	IQR
**Serum IgA antibody levels specific for LPS**				
**Day 0**	3	3–7.2	4	3–7.9
**Day 7**	94∧	19.8–231.5	3.1*	3–7.4
**Day 14**	103∧	23.9–253.5	3.4*	3–7.4
**Serum IgG antibody levels specific for LPS**				
**Day 0**	6300	3620–16560	6925	3950–10762.5
**Day 14**	66650∧	31075–123900	7680*	4170–11500
**Day 28**	55700	25450–106800	8175	4402.5–12437.5

Data from one* and two∧ subjects missing.

In the vaccine group, median LPS specific IgG antibody levels were 66650 (IQR; 31075–123900) units/ml and 55700 (IQR; 25450–106800) units/ml on day 14 and 28, respectively, compared to median baseline levels of 6300 (IQR 3620–16560) units/ml. On day 14, the 91 immune responders in the M01ZH09 group (Table 3) showed a median 8.18 (IQR; 3.57–20.68) fold increase in serum IgG antibodies relative to baseline.

Forty-two out of 44 eligible subjects provided samples for the ELISPOT assay on day 7. All baseline ELISPOT samples were negative (defined as <4 ASC per 10^6^ PBMC). On day 7, 28 (100%) of 28 M01ZH09 subjects who provided samples showed a positive ELISPOT response compared to none (0%) of the 14 evaluable subjects in the placebo group. Sixteen (57%) of 28 M01ZH09 recipients displayed results of >100 spots per 10^6^ PBMC and among the remaining 12 vaccine subjects numbers of spots ranged from 8 to 128 per 10^6^ PBMC. The median number of spots in the M01ZH09 recipients was >100 (IQR 46.5->100) spots per 10^6^ PBMC, as counting stopped above 100 spots, this was recorded as ” too many spots to be counted”. All 14 placebo recipients showed <4 spots per 10^6^ PBMC, this was recorded as “too few spots to be counted.”

There was strong correlation between the results of the IgA ELISA and the IgA ELISPOT assays on day 7. Twenty-eight (100%) of 28 M01ZH09 recipients showed a positive immune response and 14 (100%) of 14 placebo recipients showed a negative response in both assays.

## Discussion

### Interpretation

This is the first evaluation of a novel oral typhoid vaccine in school children in an endemic country. *S.* Typhi (Ty2 *aroC* ^−^ *ssaV* ^−^) ZH9 (contained in M01ZH09) is characterised by two well defined deletion mutations, one in an aromatic amino acid biosynthesis pathway gene and one in a functional gene of the type III secretion system encoded by SPI-2 [9]. A single dose of 5×10^9^ CFU of the vaccine strain was well tolerated and had an acceptable safety profile. There were no serious adverse events, no withdrawals due to adverse events and none of the subjects experienced bacteraemia.

In general, adverse events were mild. Similar proportions of subjects, 26% (26 of 101) in the candidate vaccine group and 22% (11 of 50) in the placebo group reported adverse events during the 28 day follow up period (*p* = 0.691). The overall number of adverse events tended to be higher in the M01ZH09 group, especially those classified as gastrointestinal disorders, nervous system disorders and investigations.

There was one severe related adverse event in this trial, a high fever of 39.0°C which occurred on day 0 after vaccination in a subject who had a pre-dose elevated white blood count (18.2×10^9^/L) and might have suffered from an underlying infection. One other subject vomited after drinking approximately half of the vaccine dose, this subject was found to have a positive stool culture for non-typhoid *Salmonella* on day 0.

*S.* Typhi was isolated from the stools of one placebo recipient on day 2 after vaccination which was later identified as *S.* Typhi (Ty2 *aroC* ^−^ *ssaV* ^−^) ZH9 by PCR analysis. The previous stool cultures and all following stool cultures of this subject up to day 14 were negative. After a thorough check which included the randomisation codes and vaccination paperwork, the possibility that the subject received M01ZH09 by error was excluded. The subject also did not display any positive results in the immunogenicity assays. It was concluded that the most likely cause for isolating *S.* Typhi (Ty2 *aroC* ^−^ *ssaV* ^−^) ZH9 in the stools of a placebo recipient was the mislabelling or mismatch of stool samples.

The candidate vaccine elicited a positive immune response in 97% (98/101) of the M01ZH09 recipients by ELISA and in 100% (28/28) of M01ZH09 recipients who were evaluable by ELISPOT assay. In conclusion M01ZH09 was safe and immunogenic in Vietnamese children.

### Generalisability

The observed safety and immunogenicity profile of the candidate typhoid vaccine in children compares favourably to that seen in Western adult volunteers. M01ZH09 has been tested so far up to a nominal dose level of 5×10^9^ CFU in nine UK volunteers [9] and 80 US volunteers [10], [11]. Immunogenicity results from previously published M01ZH09 trials used a 4 fold or higher increase in LPS specific IgG antibody levels as definition of a positive immune response in the endpoint titre ELISA and seroconversion rates were 50% (8/16 subjects) [10] and 77.4% (24/31 subjects) [11]. In this study, allowing for these different cut-offs, the magnitude of the immune response seen in the children was approximately 30 fold and 10 fold increase of median levels of LPS specific IgA and IgG antibodies, respectively. Furthermore. the median number of ASCs producing LPS specific IgA antibodies, a measure for priming of the mucosal immune system, was greater than 100 per 10^6^ PBMC in this trial, this compares favourably to an arithmetic mean of 118 ASC/10^6^ PBMC seen in a previous M01ZH09 trial in adults [10] and a geometric mean of 119 ASC/10^6^ PBMC (producing IgA and IgG) seen in American volunteers who received 4 doses of the licensed Ty21a typhoid vaccine at a dose of 2−6×10^9^ CFU [15].

This is encouraging as one major concern for the development of many oral vaccines has been their reduced immunogenicity when tested in developing country populations compared to Western volunteers [12], [16]. For oral vaccines a brisk colonisation of the intestine is necessary to become immunogenic, it might be possible that drug resistant commensals, bacterial overgrowth, enteric viruses or helminths interfere with the colonisation of the new vaccine [17]. In this study, 51% (51/100) of vaccine recipients shed *S.* Typhi (Ty2 *aroC* ^−^ *ssaV* ^−^) ZH9 in stools after vaccination, one subject excreted the vaccine strain on day 3, but no shedding was observed on day 4 and beyond. In Western adult volunteers shedding of *S.* Typhi in stools was reported for slightly longer durations and ranged from 1–6 days and 1–7 days in a small number of volunteers, respectively [9], [11].

### Generalisation

Typhoid fever is still a major health problem in developing countries, with high incidence [1], [18] and high rates of antimicrobial drug resistance, especially in Asia [4], [18]. The World Health Organisation recommends the immunisations of school and preschool children in endemic areas, especially where drug resistant typhoid fever is prevalent as well as in epidemic situations [5], [19]. M01ZH09 is a promising novel oral one dose typhoid vaccine and large trials are necessary to evaluate vaccine efficacy. If protection from typhoid fever is demonstrated, M01ZH09 may facilitate large vaccination campaigns due to its simpler logistic and broader acceptance from children.

## Supporting Information

Protocol S1 Trial protocol. The trial protocol is as a true and correct copy of the original document (PDF version) minus redacted lines (personal information, names and telephone numbers of employees have been removed to maintain their confidentiality). No part in the content of the trial protocol with the exception of the vaccine excipients has been redacted.
(0.23 MB PDF)

Checklist S1 CONSORT Checklist.
(0.05 MB DOC)
