# Supplementary material for: A Randomised Trial Evaluating the Safety and Immunogenicity of the Novel Single Oral Dose Typhoid Vaccine M01ZH09 in Healthy Vietnamese Children
Source: PLoS One. 2010 Jul 26;5(7):e11778. doi: 10.1371/journal.pone.0011778 (PMC2909895; doi:10.1371/journal.pone.0011778)
Supplement: Protocol S1 — Trial protocol. The trial protocol is as a true and correct copy of the original document (PDF version) minus redacted lines (personal information, names and telephone numbers of employees have been removed to maintain their confidentiality). No part in the content of the trial protocol with the exception of the vaccine excipients has been redacted. (0.23 MB PDF) [file pone.0011778.s001.pdf]

CLINICAL STUDY PROTOCOL

Study Number MS01.08

Formerly Microscience Ltd

PROTOCOL

A placebo controlled, single-blind, single oral dose study to determine the safety and immunogenicity of M01ZH09 typhoid vaccine (oral live *S. typhi* (Ty2 *aroC? ssaV?*) ZH9) in healthy paediatric subjects, aged 5 to 14 years inclusive, of Vietnamese origin.

PROTOCOL NUMBER MS01.08 V2

SPONSOR

Emergent Product Development UK Limited
(formerly Microscience Ltd)
540-545 Eskdale Road
Winnersh Triangle
Wokingham
Berkshire
RG41 5TU
United Kingdom

Tel: + 44 (0)118 944 3300
Fax: + 44 (0)118 944 3302

CONTACT DETAILS

Study Manager

Medical Monitor

Emergency (out of hours) Contact

CONTACT FOR SERIOUS ADVERSE EVENT REPORTING

AKOS Ltd.
The Coach House
The Grove
Pipers Land,
Harpenden
Herts, AL5 1AH

Telephone: 01582 766339
Fax: 01582 764327

Fax Number for SAE Reporting:

E-Mail

CONFIDENTIALITY STATEMENT

EMERGENT PRODUCT DEVELOPMENT UK LIMITED

PROTOCOL APPROVAL

AUTHOR

I have written the contents of this protocol MS01.08 Final V2 17 January 2007

______________________________ Date:______________________

Author signature

EMERGENT PRODUCT DEVELOPMENT UK LIMITED

PROTOCOL APPROVAL

MEDICAL MONITOR

I have reviewed the contents of this protocol MS01.08 V2 and I consider it appropriate to administer the vaccine to healthy paediatric subjects according to the agreed protocol MS01.08 V2

_______________________________ Date: _______________________

Medical Monitor signature

EMERGENT PRODUCT DEVELOPMENT UK LIMITED

PROTOCOL APPROVAL

STATISTICIAN

The statistical analyses described are appropriate for study MS01.08 V2

______________________________ Date:______________________

Statistician signature

Statwood Ltd
Bevan House
Bancroft Court
Hitchin
Herts
SG5 1LH

EMERGENT PRODUCT DEVELOPMENT UK LIMITED

PROTOCOL APPROVAL

SPONSOR STATEMENT

Emergent Product Development UK Ltd (the Sponsor) approves the contents of protocol MS01.08 V2 and agrees that it will arrange for the supply of the investigational products described in the protocol and undertakes to report adverse events to the relevant authorities in compliance with the regulations. It further agrees to inform the Investigator of any information that would place the subjects at risk by their continuing participation in the study.

______________________________ Date: ______________________

Sponsor signature

INVESTIGATOR STATEMENT OF AGREEMENT

Having considered the information available on the use of freeze-dried live attenuated (and reconstituted) *S. typhi* (Ty2 aroC? ssaV?) ZH9 oral live typhoid vaccine, I consider it safe and ethically justified to administer the vaccine to healthy paediatric subjects according to the agreed protocol MS01.08 V2. In addition, I agree to conduct the study and report and adverse events in accordance with the terms of the study protocol and that it will be conducted according to the ICH Good Clinical Practice Standard.

Jeremy Farrar

Principal Investigator

_______________________________ _________________

Investigator signature Date:

Principal Investigator
Jeremy Farrar, Director
Oxford University Clinical Research Unit
Hospital for Tropical Diseases
Ho Chi Minh City, Viet Nam
Tel:
Fax:
E-mail:

Tran Tinh Hien

Principal Investigator

_______________________________ _________________

Investigator signature Date:

Principal Investigator
Vice-Director Hospital for Tropical Diseases
The Hospital for Tropical Diseases,
190 Ben Ham Tu, Quan 5,
Ho Chi Minh City, Viet Nam
Tel:
Fax:

Study Centre

Oxford University Clinical Research Unit
Hospital for Tropical Diseases
Ho Chi Minh City, Viet Nam

TABLE OF CONTENTS

2.7 Risks and Benefits 15
Section 4.5 Blinding 16
Section 4.7 Duration of Subject Participation 16
6.8.2 Visit 1: Day of Dosing/ Day 0 16
6.8.2 Visit 1: Day of Dosing/ Day 0 16
6.8.2 Visit 1: Day of Dosing/ Day 0 16
8.1.2 Serious Adverse Event 17
8.2 Collection, Recording and Reporting of Adverse Events 17
1 PROTOCOL SUMMARY 18
1.1 Study Schedule 23
2 BACKGROUND INFORMATION 25
2.1 Typhoid Disease 25
2.2 Investigational Product 27
2.3 Non-Clinical Summary 28
2.4 Clinical Summary 29
2.5 Rationale for Dose 33
2.6 Study Population 33
2.7 Risks and Benefits 33
2.8 Study Treatment 34
2.9 Compliance Statement 35
3 STUDY PURPOSE, OBJECTIVES AND ENDPOINTS 36
3.1 Purpose 36
3.2 Objective 36
3.3 Safety Endpoints 36
3.3.1 Primary 36
3.3.2 Secondary 36
3.4 Immunogenicity Endpoints 36
4 STUDY DESIGN 38
4.1 Summary of Study Design 38
4.2 Rationale for Study Design 38
4.3 Study Population 38
4.4 Rationale for Dose 38
4.5 Blinding 38
4.6 Study Treatments 39
4.7 Duration of Subject Participation 39
4.8 Stopping Rules 40
4.8.1 Study as a Whole 40
4.8.2 Individual Subjects 42
4.9 Randomisation Codes and Breaking the Code 42
5 SELECTION AND WITHDRAWAL OF SUBJECTS 43
5.1 Inclusion Criteria 43
5.2 Exclusion Criteria 43
5.3 Withdrawal Criteria 44
5.4 Subject Replacement 45

5.5 Follow -up of Withdrawn Subjects 45
6 TREATMENT OF SUBJECTS 46
6.1 Study Products 46
6.2 Method of Administration of Each Dose 46
6.3 Packaging and Labelling of Study Products 47
6.4 Storage and Drug Accountability of Study Products 47
6.5 Other Materials 48
6.6 Concomitant Medication 48
6.7 Compliance 49
6.8 Visits and Procedures 49
6.8.1 Screening period (Day –28 to Day –1) 49
6.8.2 Visit 1: Day of Dosing/ Day 0 50
6.8.3 Visit 2: Day 1 51
6.8.4 Visits 3, 4, 5, 6 and 7: Days 2, 3, 4, 5 and 6 51
6.8.5 Visit 8: Day 7 52
6.8.6 Visit 9, 10, 11, 12, 13 and 14: Days 8, 9, 10, 11, 12 and 13 52
6.8.7 Visit 15: Day 14 53
6.8.8 Visit 16: Day 28 53
6.8.9 Specific Safety Reviews at Each Visit 54
6.8.10 Unscheduled Visits 54
7 IMMUNOGENICITY, SAFETY AND LABORATORY ASSESSMENTS 55
7.1 Assessments of Immunogenicity 55
7.1.1 Measure of Antibody Secreting Cells by ELISPOT 55
7.1.2 Analysis of LPS Specific Immunoglobulin G by the ELISA Assay 55
7.1.3 Analysis of LPS Specific Immunoglobulin A by the ELISA Assay 56
7.2 Assessment for Safety 56
7.2.1 Laboratory Tests 56
7.2.2 Physical Examination 57
7.2.3 Vital Signs 58
7.2.4 Diary Card 58
8 ADVERSE EVENTS 59
8.1 Definitions 59
8.1.1 Adverse Event 59
8.1.2 Serious Adverse Event 59
8.1.3 Severity Assessment Definitions 60
8.1.4 Relationship to Study Product Assessment Definitions 60
8.1.5 Outcome Categories and Definitions 60
8.1.6 Fever Definition 61
8.2 Collection, Recording and Reporting of Adverse Events 61
8.3 Follow-up of Adverse Events 61
8.4 Safety Committee 61
8.5 Pregnancy 61
8.6 Overdose 62
9 STATISTICS 63
9.1 Sample Size Calculation 63
9.2 Statistical Methods 63

9.2.1 General Points 63
9.2.2 Admission Profile 63
9.2.3 Study Populations 63
9.2.4 Safety Endpoints 63
9.2.5 Immunogenicity Endpoints 64
9.3 Methods of Analysis for Safety Variables 64
9.4 Methods of Analysis for Immunogenicity Endpoint 65
10 DIRECT ACCESS TO SOURCE DATA/ DOCUMENTS 66
11 QUALITY CONTROL AND QUALITY ASSURANCE 66
12 ETHICS 67
12.1 Informed Consent Form for Study Subjects 67
12.2 Independent Ethics Committee 67
12.3 Regulatory Authorities 68
13 DATA HANDLING AND RECORD KEEPING 68
13.1 Data Entry 68
13.2 Data Coding 68
13.3 Audit Trail 68
13.4 Data Validation and Checks 68
14 FINANCE AND INSURANCE 69
15 REPORTS AND PUBLICATIONS 69
16 ARCHIVING 70
17 REFERENCES 70

TERMS AND ABBREVIATIONS

°C	Degrees Celsius
™	Trademark
μg	Microgram
ADR	Adverse Drug Reaction
AE	Adverse Event
ALT	Alanine aminotransferase
ASC	Antibody Secreting Cells
AST	Aspartate aminotransferase
BP	Blood Pressure
CFR	Code of Federal Regulations
CFU	Colony Forming Units
CRF	Case Report Form
CV	*Curriculum Vitae*
ELISA	Enzyme Linked ImmunoSorbant Assay
ELISPOT	Enzyme Linked ImmunoSpot Assay
g	Gram
GCP	Good Clinical Practice
gGT	Gamma-glutamyl transferase
HIV	Human Immunodeficiency Virus
IB	Investigator’s Brochure
ICH	International Conference on Harmonisation
IFNγ	Gamma-interferon
IgA	Immunoglobulin A
IgG	Immunoglobulin G
IMP	Investigational Medicinal Product
IRB	Institutional Review Board
ITT	Intent-to-treat
LPS	Lipopolysaccharide
MALT	Mucosal Associated Lymphoid Tissue
MedDRA	Medical Dictionary for Regulatory Activities

mg	Milligram
MIC	Minimum Inhibitory Concentration
mL	Millilitre
PBMC	Peripheral Blood Mononuclear Cells
PP	Per protocol
RES	Reticulo-endothelial system
SAE	Serious Adverse Event
SAP	Statistical Analysis Plan
SD	Standard Deviation
SDV	Source Data Verification
SPI-2	*Salmonella* Pathogenicity Island 2
UK	United Kingdom
w/v	Weight per Volume

Amendments

Protocol Amendment 1

Date of Preparation of Amendment: 17 January 2007

Reason for Amendment

The reasons for this amendment are given below:

a. The sponsor has delegated the management of SAE reporting to the specialised SAE Management and safety surveillance organisation, AKOS Ltd.

b. The statistician for the trial has now changed due to personnel changes at Statwood the statistical group contracted to conduct the data management and statistical analysis.

c. Plans to conduct a long term follow-up (MS01.10) in these subjects have been discontinued.

d. Minor administrative and typographic amendments have also been made to aid understanding and for internal consistency.

These changes do not affect the procedures that the subjects will undergo with respect to this protocol and have no impact of the written information provided to the subject.

Specific Changes

Location	Original Text	New Text
Page 3	None- New text added.	**CONTACT FOR SERIOUS ADVERSE EVENT REPORTING**<br>AKOS Ltd.<br>The Coach House<br>The Grove<br>Pipers Land,<br>Harpenden<br>Herts, AL5 1AH<br><br>Telephone: 01582 766339<br>Fax: 01582 764327<br>**Fax Number for SAE Reporting:**<br><br>E-Mail
Page 7		
2.7 Risks and Benefits	…"The anticipated risks of the study are in line with the side effect profile seen to date with M01ZH09. The most likely adverse effects are flatulence, abdominal	…"The anticipated risks of the study are in line with the side effect profile seen to date with M01ZH09. The most likely adverse effects are flatulence,

	cramps/discomfort, loose/unformed stools, headache, nausea, vomiting, chills/rigors, minor elevations in body temperature (to < 38°C) and mild muscle and joint pain in subjects receiving the active vaccine."…	abdominal cramps/discomfort, loose/unformed stools, headache, nausea, vomiting, chills/rigors, minor elevations in body temperature (**up to 38**°C) and mild muscle and joint pain in subjects receiving the active vaccine."…
Section 4.5 Blinding	"The investigational products will be blinded by dispensing the dosing solutions in unmarked beakers and will be controlled and dispensed by the clinic pharmacist according to the pre-prepared randomisation lists. Hence the investigator will be essentially blinded in that knowledge of the product supplied to the subjects will remain unknown. "…	"Active vaccine and matching placebo will be packaged identically but with a unique sequential number. This number will enable identification of the contents in case of emergency when used with the randomisation code break. Preparation of both active and placebo doses will be identical. Hence the investigator and his staff will be essentially blinded in that knowledge of the product supplied to the subjects will remain unknown."
Section 4.7 Duration of Subject Participation	"six months. All subjects will be invited to take part in a long-term follow-up study MS01.10."	"six months.
6.8.2 Visit 1: Day of Dosing/ Day 0	• Enquire about and record any changes in concomitant medication since screening.	• Enquire about and record any changes in concomitant medication since screening<br>**If the subject has received antibiotic therapy within 14 days of this visit, arrange for the subject to return for dosing if this is possible within 28 days of the screening visit. If not, please withdraw the subject from the study.**<br>**Subjects who are withdrawn at Visit 1, under these circumstances and cannot be dosed within 28 days of the Screening Visit may be re-screened. Under these circumstances the subject must be re-consented.**
6.8.2 Visit 1: Day of Dosing/ Day 0	• Determine and record vital signs (blood pressure, temperature and pulse).	• Determine and record vital signs (blood pressure, temperature and pulse).<br>**If oral temperature is 38°C or more, arrange for the subject to return for dosing if this is possible within 28 days of the screening visit. If not, please withdraw the subject from the study.**<br>**Subjects who are withdrawn at Visit 1, under these circumstances and cannot be dosed within 28 days of the Screening Visit may be re-screened. Under these circumstances the subject must be re-consented**
6.8.2 Visit 1: Day of Dosing/	• Make an appointment for Study Day 1	• Make an appointment for Visit 2/

Day 0	and instruct the subject to bring a stool sample to the visit.	Day 1 and instruct the subject to bring a stool sample to the visit.
8.1.2 Serious Adverse Event	…"reported to Emergent Product Development UK Ltd following the SAE reporting procedure. If the blood culture grows the M01ZH09 vaccine strain or if the cause of the bacteraemia is undetermined Emergent Product Development UK Ltd will report the event as a serious, unexpected, related, adverse event."..	…"reported to **Akos Ltd.** following the SAE reporting procedure. If the blood culture grows the M01ZH09 vaccine strain or if the cause of the bacteraemia is undetermined **Akos Ltd.** will report the event as a serious, unexpected, related, adverse event."..
8.2 Collection, Recording and Reporting of Adverse Events	"All SAEs, whether or not they are judged to be related to the study drug, should be reported, by the Investigator, to Emergent Product Development UK Ltd, upon learning of the SAE. SAEs must be reported within 24 hours of knowing about the event or, ideally, within 24 hours of the occurrence of the event. The Investigator must complete the Emergent Product Development UK Ltd SAE form with as much information as is available at the time of completion and fax the form immediately to the number provided. The Study Monitor, the Medical Monitor, or a representative of Emergent Product Development UK Ltd, may follow up by requesting clarification or additional information on the SAE, from the Investigator."…<br><br>…"It is the responsibility of the Investigator (with the assistance of Emergent Product Development UK Ltd) to report any serious, unexpected ADRs to the Ethics Committee in accordance with ICH guidelines and local requirements."…	"All SAEs, whether or not they are judged to be related to the study drug, should be reported, by the Investigator, to **Akos Ltd.**, upon learning of the SAE. SAEs must be reported within 24 hours of knowing about the event or, ideally, within 24 hours of the occurrence of the event. The Investigator must complete the **Akos Ltd.** SAE form with as much information as is available at the time of completion and fax the form immediately to the number provided. **Akos Ltd.,** the Study Monitor, the Medical Monitor, or a representative of Emergent Product Development UK Ltd, may follow up by requesting clarification or additional information on the SAE, from the Investigator."…<br><br>It is the responsibility of the Investigator (with the assistance of Emergent Product Development UK Ltd and/or **Akos Ltd.** to report any serious, unexpected ADRs to the Ethics Committee in accordance with ICH guidelines and local requirements."…
8.3 Follow-up of Adverse Events	…"The Investigator must forward follow-up information on SAEs to Emergent Product Development UK Ltd within 5 days of obtaining the follow-up information. Only changed or new information should be supplied."…	…"The Investigator must forward follow-up information on SAEs to **Akos Ltd.** within 5 days of obtaining the follow-up information. Only changed or new information should be supplied."…

1 PROTOCOL SUMMARY

**Protocol Number**	MS01.08 V2
**Title of the Study**	A placebo-controlled, single-blind, single oral dose study to determine the safety and immunogenicity of M01ZH09 typhoid vaccine (oral live *S. typhi* (Ty2 *aro*C? *ssa*V?) ZH9) in healthy paediatric subjects, aged 5 to 14 years inclusive, of Vietnamese origin.
**Sponsor**	Emergent Product Development UK Ltd (formerly Microscience Ltd)
**Phase of Development**	Phase II
**Regulatory Status**	IND No. BB-IND 10176
**STUDY OBJECTIVES**	
**Primary**	To evaluate the safety and immunogenicity of M01ZH09 typhoid vaccine (oral live *S. typhi* (Ty2 *aro*C? *ssa*V?) ZH9) at a nominal dose of 5 x 10^9 colony forming units (CFU) in healthy paediatric subjects from Viet Nam.
**Study Design**	A single centre, single-blind, placebo-controlled, randomised, parallel group study.
**SUBJECT POPULATION**	
**Number of Subjects**	150 subjects will be randomised and receive M01ZH09 oral typhoid vaccine or placebo in a 2:1 ratio (100 active : 50 placebo)
**Number of Groups**	2 groups of subjects.<br>1. 100 subjects will receive M01ZH09<br>2. 50 subjects will receive placebo.<br>At least 70% of the subjects in each group will be aged 5 to 10 years inclusive.
**Status/ Sex/ Age**	Healthy male and female paediatric subjects aged 5 to 14 inclusive.
**Study Groups**	Group 1. Subjects will receive a nominal dose of 5 x 10^9 CFU of M01ZH09 typhoid vaccine. Vaccine will be administered in a bicarbonate solution containing 1.75% (w/v) sodium bicarbonate, 1.1% (w/v) ascorbic acid and 0.02% (w/v) aspartame. The solution is prepared from bicarbonate tablets using drinking water.<br>Group 2. Subjects will receive placebo administered in a bicarbonate solution containing 1.75% (w/v) sodium bicarbonate, 1.1% (w/v) ascorbic acid and 0.02% (w/v) aspartame. The solution is prepared from bicarbonate tablets using drinking water.<br>Subjects aged 5 to 10 years will receive the vaccine or placebo in

	75 mL of bicarbonate solution. Subjects aged 11 to 14 years will receive the vaccine or placebo in 150 mL of bicarbonate solution.
**Study Medication**	*S. typhi* (Ty2 *aro*C? *ssa*V?) ZH9 at a nominal dose of 5 x 10^9 CFU.<br><br>M01ZH09 is supplied as a freeze-dried formulation of the vaccine strain, plus excipients, in single dose vials. The placebo is supplied as a freeze-dried formulation of vaccine excipients only, in single dose vials. Effervescent bicarbonate tablets containing, sodium bicarbonate, ascorbic acid and aspartame will also be supplied.
**Dosing Regimen**	Single oral dose.
**Inclusion Criteria**	1. Healthy paediatric subjects aged 5 to 14 years inclusive, of Vietnamese origin, who are able and willing to take part in the trial and whose parents or guardians give written permission for their child's participation, following a detailed explanation of the study.<br><br>2. Subjects who will be available for the duration of the study and available for scheduled and potential additional visits.
**Exclusion Criteria**	1. Subjects with any clinically significant medical or psychiatric condition or clinically significant abnormal serum biochemistry or haematology results that, in the opinion of the Investigator, preclude participation in the study.<br><br>2. Subjects whose body weight is under 17kg (5-10 year olds), or under 27kg (11-14 year olds)<br><br>3. Female subjects who are pregnant (confirmed with urinary pregnancy test) or breast-feeding, or of childbearing potential and unwilling to use a reliable method of contraception (oral contraceptives, barrier method with spermicidal preparation or abstinence) throughout the study period.<br><br>4. Subjects who have a known hypersensitivity to two or more of the following antibiotics; ciprofloxacin, azithromycin or trimethoprim-sulfamethoxazole or have used antibiotics/antibacterials within 14 days prior to administration of study medication.<br><br>5. Subjects who have a known hypersensitivity to any component of the vaccine or bicarbonate solution used in this study, including subjects with phenylketonuria or those who have ever experienced anaphylactic shock after any vaccination.<br><br>6. Subjects who received Vivotif, either as a licensed or investigational product, in the last 10 years or any other vaccine against *S. typhi*, whether licensed or investigational, in the last 5 years, or who have ever suffered from typhoid fever.<br><br>7. Subjects with direct contact with patients in special care units

	or immuno-compromised individuals.<br>8. Subjects who have a positive bacterial culture of their faecal sample, obtained at the screening visit, for any *Salmonella* species.<br>9. Subjects with a known impairment of immune function including acquired immune deficiency syndrome or those receiving (or having received in the 6 months prior to screening) cytotoxic drugs, immunosuppressive therapy (including systemic corticosteroids).<br>10. Subjects who are HIV positive or have family members who are HIV positive. ('Family members' includes immediate and extended family living in the same residential unit, or relations with whom the subject has regular and frequent contact).<br>11. Subjects with a significant acute febrile illness (body temperature of 38.0ºC or more) at time of dosing.<br>12. Subjects who have chronic diseases: Chronic diseases will include all autoimmune and immuno-compromising conditions and any other chronic condition which, at the judgement of the Investigator, may put the subject at higher risk of side effects from the study vaccine. Conditions in the latter category might include unexplained anaemia, hepato-biliary disease, uncontrolled hypertension, subjects with prosthetic joints or heart valves etc.<br>13. Subjects with a current problem, based on history, of substance abuse or with a history of substance abuse that, in the opinion of the investigator, might interfere with participation in the study.<br>14. Subjects who are currently involved in a clinical study, have taken an investigational drug or have received investigational or licensed vaccines in the 4 weeks before screening, or anticipate receiving a vaccine, other than study medication, during the first 4 weeks, post vaccination, of the study.
**Study Conduct/ Procedures**	Following completion of the screening assessment, subjects who satisfy the study entry criteria will be randomised to one of the two treatment groups (active or placebo) and will receive medication on Day 0. Subjects will then return to the investigative site every day until Day 14, and then again on Day 28, for assessment of safety and immunogenicity and to provide blood, urine and stool samples as required (see schedule of events). Subjects will record their oral temperatures on a Diary Card, twice daily, for the first 14 days following dosing and will return to the clinic for additional visits should they develop fever or other signs

	potentially indicating a bacteraemia.
**Safety Endpoints**	Primary<br><br>The proportion of subjects reporting Serious Adverse Events attributed to the study medication.<br><br>Secondary<br><br>Proportion of subjects;<br><br>• Experiencing an elevated body temperature of 38.5°C or greater, in the 14 days following dosing, attributed to study medication.<br><br>• Demonstrating persistent (after Day 7) faecal shedding of *S. typhi* (Ty2 *aro*C?*ssa*V?) ZH9.<br><br>• Withdrawn from the study due to adverse events, including bacteraemia attributed to study medication.<br><br>• With clinically significant changes in laboratory parameters, from Day 0 to any time post dosing, which are attributed to study medication.
**Immunogenicity Endpoints**	Assessment of the immune response will be made by determining the proportion of subjects who;<br><br>• Develop a positive immune response* to *S. typhi* lipopolysaccharide (LPS) as assessed by an increase in *S. typhi* LPS specific IgG at either Day 14 or Day 28.<br><br>• Develop a positive immune response* to *S. typhi* LPS as assessed by an increase in S. *typhi* LPS specific IgA at either Day 7 or Day 14.<br><br>• At Day 7, have = 4 antibody secreting cells (ASCs) per 10^6 peripheral blood mononuclear cells (PBMC), secreting IgA specific for *S. typhi* LPS detected by ELISPOT assay.<br><br>*The definition of a ‘positive immune response’ will be determined following the completion of assay development.
**Statistical Analyses**	The primary outcome measure in this study will be the proportion of subjects with a positive immune response (a positive serum IgG and/or a positive serum IgA response and/or an increase in the number of PBMC secreting IgA specific for *S.typhi* detected by ELISPOT assay). The proportion of subjects who satisfy the immunogenicity endpoint will be compared between the vaccine and placebo groups. The treatment difference and associated 95% confidence interval will be presented.<br><br>The number and percentage of subjects with an immune response will be presented along with the two-sided 95% confidence

	interval for each treatment group.<br>In addition, the number and percentage of subjects with defined immune response will be presented by type of assay.<br>For the safety endpoints, the proportion of subjects that have a SAE, attributed to the study medication, will be compared between groups. The proportion of subjects with symptomatic elevation in body temperature, persistent faecal shedding and clinically significant changes in laboratory parameters will be compared between groups.

1.1 Study Schedule

**Visit**	**Screen**	**1**	**2**	**3**	**4**	**5**	**6**	**7**	**8**	**9**	**10**	**11**	**12**	**13**	**14**	**15**	**16**
**Day**	**-28 to -1**	**0**	**1**	**2**	**3**	**4**	**5**	**6**	**7**	**8**	**9**	**10**	**11**	**12**	**13**	**14**	**28**
Informed Permission and Assent	X																
Inclusion/exclusion criteria	X	X[1]															
Demography/medical history	X	X															
Medication history (particularly antibiotics)	X	X															
Physical examination	X																X
Vital signs[2]	X	X	X	X	X	X	X	X	X	X	X	X	X	X	X	X	X
Blood draw for HIV	X																
Issue digital thermometer		X															
Issue Diary card		X															
Review data on Diary Card			X	X	X	X	X	X	X	X	X	X	X	X	X	X	
Review adverse events		X	X	X	X	X	X	X	X	X	X	X	X	X	X	X	X
Review splenomegaly	X	X	X	X	X	X	X	X	X	X	X	X	X	X	X	X	X
Review changes in concomitant medications		X	X	X	X	X	X	X	X	X	X	X	X	X	X	X	X
Dosing		**X**															
**Laboratory – safety:**																	
Blood draw for haematology and biochemistry	X	X							X							X	X
Urine pregnancy test, if applicable[3]	X	X															X
Urinalysis (dip stick)[4]	X	X							X							X	X
Stool culture[5]	X	X	X	X	X	X	X	X	X	X	X	X	X	X	X	X	
**Laboratory – immunogenicity:**																	
Blood draw for IgG (serum ELISA) assay		X														X	X
Blood draw for IgA (serum ELISA) assay		X							X							X	
PBMC Blood draw for ELISPOT assay[6]		X							X								

1. On receipt of screening visit laboratory results, check that the subject is still eligible for study entry.
2. Oral temperature, blood pressure and pulse rate conducted prior to dosing on Day 0 and then on each subsequent study visit. Subjects will take their own temperature or have their temperature taken, daily, in the morning when they get up and when they go to bed for Days 1 through 14 and record the reading on their Diary Cards. (On day of dosing, blood pressure and pulse will be taken 30, 60 and 90 minutes post dose).
3. For all female subjects aged 11 years and older.

4. If urine dipstick test shows presence of nitrites and leucocyte esterase, a fresh urine sample will be sent for microscopy, culture and sensitivity. Additionally at V8, 15 and 16 cultures to determine if *S.typhi* (Ty2 *aro*C?*ssa*V?) ZH09 is present will also be performed

5. Stool samples will be sent for culture of Salmonella spp and in addition at V2-15 to determine if vaccine strain *S.typhi* (Ty2 *aro*C?*ssa*V?) ZH09 is present.
6. ELISPOT assay to be performed only on subjects aged at least 11 years.

2 BACKGROUND INFORMATION

2.1 Typhoid Disease

Typhoid fever is an acute, generalised infection of the reticuloendothelial system (RES), intestinal lymphoid tissue and gall bladder caused by *Salmonella enterica* serovar Typhi (*S. typhi)*. It is restricted to causing human disease and therefore humans (chronic carriers) serve as the epidemiological reservoir of infection.

The disease is spread orally through the ingestion of contaminated food and water. *S. typhi* is highly invasive and, once ingested, passes through the intestinal mucosa to reach the lamina propria. Shortly after this a primary bacteraemia takes place, during which the *S. typhi* are filtered from the circulation by fixed phagocytic cells of the RES. As a result of this primary bacteraemia, the pathogen rapidly attains an intracellular haven throughout the organs of the RES where it resides during the incubation period (usually 8 to 14 days) until the onset of clinical typhoid fever. Clinical illness is accompanied by a sustained secondary bacteraemia.

In populations in developing countries that are not served with treated water supplies and sanitation to remove human waste, typhoid fever is endemic. There are approximately 17 million cases of typhoid fever reported globally every year, but as not all cases are reported it has been estimated that the true figure may be as high as 30 million cases globally. There are 600,000 deaths per annum worldwide attributed to typhoid fever, nearly all of these occurring in developing countries. Some three-quarters of the global burden occurs in Asia; the bulk of which is borne by pre-school and school-aged children. In South Viet Nam, typhoid fever remains a significant intestinal infection. Between 1990 and 1993, among 15 districts in the South of the country, a total of 3,853 to 9,179 cases per year were registered. Of these there were from 8 to 31 deaths. In 1993 an epidemic of typhoid fever broke out in the An Minh district (territory of Kien Giang, South Viet Nam), affecting 3,049 people and causing two deaths. Of the 574 blood samples analysed, 266 strains of *S. typhi* were isolated (Nguyen, Ha Ba and Nguyen 1993). Viet Nam, particularly in the southern provinces, experienced a greater than six-fold increase in reported cases of typhoid fever from 1990 (4,859 cases) to 1995 (30,901). Most cases (90%) were reported from the southern region which consists of 17 provinces that comprise about 39% of the total population of Viet Nam. Typhoid is a significant disease in pre-school and school children in Viet Nam.

Typhoid fever has become difficult and expensive to treat. In southern Viet Nam, about 90% of *S. typhi* isolates are now resistant to multiple antibiotics including chloramphenicol, ampicillin and co-trimoxazole. More recently, 76% of blood culture isolates of *S. typhi* were reported to be resistant to nalidixic acid (Lin *et al* 2000). Over the last few years, the incidence of typhoid fever in Viet Nam has decreased but still occurs.

The increasing prevalence of *S. typhi* strains exhibiting resistance to multiple, previously effective, antibiotics has complicated the therapy of typhoid fever, and has rekindled the interest in the development of more effective typhoid vaccines.

Inactivated (heat-killed, phenol-preserved) vaccines have been used for many years and have been shown to confer moderate protection (Levine, 1994). However, they were never well accepted as public health tools because of the adverse systemic reactions that occurred with high frequency (about 25%) in the recipients of such vaccines. Two newer vaccines have now superseded the whole cell vaccines, a purified Virulence antigen (Vi) polysaccharide vaccine and an orally delivered live attenuated vaccine.

In the 1970s and early 1980s methods were developed to purify the Vi polysaccharide antigen from *S. typhi*. The purified Vi vaccine is free from contaminating Lipopolysaccharide (LPS), which is associated with systemic adverse events in injectable whole cell vaccines. Vi is an essential virulence factor and antibodies against the antigen are protective against typhoid disease. In field studies purified Vi has proved to be well tolerated (around 1 to 2% of recipients have febrile reactions) and confers efficacy of around 70% after a single dose (Klugman *et al*., 1987, Klugman *et al*., 1996; Achayra *et al*., 1987). Purified Vi vaccines (Typhim-Vi, Aventis Pasteur MSD and Typherix, SmithKline Beecham) are commercially available, have similar efficacy to that of the whole cell vaccines (Achayra *et al*., 1987) and have replaced the killed whole cell vaccines as the parenteral vaccine of choice. Like other polysaccharide vaccines, immune responses to Vi are age-related and it is not very immunogenic in children. It is also a T cell-independent antigen and it is not possible to boost the primary response. There is clearly room for improvement in terms of immunogenicity, induction of immunological memory and protective efficacy of these vaccines. Conjugated vaccines are now under development that can potentially overcome these issues. Trials of a conjugate of Vi to a non-toxic recombinant *Pseudomonas aeruginosa* exotoxin A are promising, demonstrating that this vaccine is safe and immunogenic, has more than 90% efficacy in children 2 to 5 years old, gives enhanced immunogenicity in adults and children of 5 to 14 years old (Kossaczka *et al*., 1999) and was able to elicit a booster response in children of 2 to 4 years (Lin *et al*., 2001).

S. typhi Ty21a (Vivotif Berna™, Berna) is a live attenuated *S. typhi* strain. It was attenuated using rounds of chemical mutagenesis and is Vi negative (Germanier and Furer, 1975). The exact mechanism of attenuation is unknown. This vaccine has demonstrated variable efficacy in several controlled field studies using different formulations. However, in order for the vaccine to be effective it has to be given in multiple doses (three in Europe and four in the US) and recipients have to remember to take the additional doses, and this raises a compliance issue. Once again, there is clearly room for improvement, both in terms of efficacy and convenience.

It is well established that live attenuated organisms can be highly effective vaccines, immune responses elicited can often be of greater magnitude and of longer duration than those produced by non-living antigens (Collins 1974; Hormaeche *et al*., 1990), and are often able to confer protection after a single dose. This is probably because these attenuated strains are able to mimic the early stages of natural infection. In addition, unlike killed preparations, live *Salmonella* vaccines are able to induce potent cell mediated responses (Sztein *et al*., 1994, 1995; Gonzalez *et al*., 1994), which may be connected with their ability to replicate in antigen-presenting cells such as macrophages. T cell proliferation, cytokine production, cytotoxic T cell responses and antibody-dependent cell cytotoxicity have all been elicited by live attenuated *S. typhi* vaccines (Levine and Sztein, 1996).

For the majority of pathogens, the first contact with the host is at a mucosal surface and the specific and non-specific immune mechanisms operating at these surfaces play a major role as the first line of defence. Thus, when considering new vaccine strategies to combat organisms that invade mucosal surfaces, it is important to consider priming the mucosal-associated lymphoid tissue (MALT) in order to maximize the effectiveness of vaccination. Unfortunately, parenteral vaccination is not efficient at eliciting mucosal responses as the primary requirement for such responses is contact of the immunogen directly with the mucosal surface (McGhee *et al*., 1992).

One of the most desirable ways to deliver a vaccine is via the oral route, which avoids the use of needles and reduces systemic reactogenicity. However, non–living vaccines, including *S. typhi*

vaccines (DuPont *et al*., 1971; Chuttani *et al*., 1973), are poor at eliciting either a mucosal or systemic response when given orally.

The advantage of a live *Salmonella* vaccine therefore, is that it can be given orally (which is the natural route of infection), and live *salmonellae* are known to stimulate potent humoral, cellular and secretory anti-*Salmonella* responses (Chatfield *et al*., 1993).

There has been a history of the use of live attenuated *Salmonella* vaccines as safe and effective vaccines for the prevention of salmonellosis in animals and humans. Ty21a (Vivotif Berna™) has proved to be a very successful vaccine for the prevention of typhoid fever. However, as mentioned above, the attenuation of this strain was achieved using chemical mutagenesis techniques and the basis of attenuation of the strain is not fully understood. In addition it is difficult to use chemical mutagenesis to prepare a vaccine with optimal immunogenicity.

The advent of modern molecular biology techniques, coupled with the increasing knowledge of *Salmonella* pathogenesis has led to the identification of several genes that are essential for the *in vivo* growth and survival of the organisms. This has provided new gene targets for attenuation, leading to the concept that introducing defined non-reverting mutations into selected genes known to be involved in virulence can 'rationally' attenuate future vaccine strains. This has facilitated the development of improved vaccines, particularly in terms of increasing the immunogenicity and therefore reducing the number of doses that have to be given.

This study is designed to investigate the immunogenicity and safety of a new single dose live attenuated oral vaccine against typhoid fever in healthy paediatric subjects from Viet Nam. The product, M01ZH09, contains a strain of *S. typhi* that has been rationally attenuated, by the introduction of two, independently attenuating, deletion mutations in its chromosome. The clinical studies performed to date with M01ZH09 have assessed safety, tolerability and immunogenicity in adult subjects in the USA, UK and Viet Nam. Later in the clinical development of M01ZH09, Phase III field trials will be necessary in an area where typhoid fever is endemic. Potential endemic areas for such a study exist in Viet Nam and Nepal. Field trials of a typhoid vaccine will involve children, as children under 10 years of age make up a significant proportion of the population that experience typhoid disease. The purpose of this study is to demonstrate immunogenicity and safety in a paediatric Vietnamese population prior to initiating field trials where it is anticipated that the youngest subjects that will be vaccinated will be 5 years of age.

2.2 Investigational Product

M01ZH09 is a live attenuated *S. typhi* oral vaccine, against typhoid fever, previously identified as MICRO-TY. M01ZH09 consists of a freeze-dried formulation of the vaccine strain, *S. typhi* (Ty2 *aro*C$^-$ *ssa*V$^-$) ZH9, plus excipients, supplied in a single dose vial.

S. typhi strain *S. typhi* (Ty2 *aro*C$^-$ *ssa*V$^-$) ZH9 contains defined (independently attenuating) deletions in two genes, *aro*C and *ssa*V. The *aro*C gene encodes chorismate synthase, an enzyme involved in the biosynthesis of aromatic compounds. a*ro* mutations are well described as being attenuating for *Salmonella* in humans, and the basis is presumed to be that two aromatic compounds, para-aminobenzoic acid and 2,3-dihydroxybenzoic acid are limiting for growth in mammalian tissue. The *ssa*V gene is encoded on *Salmonella* Pathogenicity Island 2 (SPI-2). SPI-2 encodes a type III secretion system and *ssa*V is a structural gene encoding part of the secretion apparatus. SPI-2 mutations are attenuating in mice, and there is evidence that this is because SPI-2

is required for survival and growth within macrophages, which normally mediate the systemic spread of the organism (Shea *et al.*, 1999). The rationale for inclusion of the *ssaV* deletion mutation is that it will prevent systemic spread of the vaccine strain in humans, and therefore prevent bacteraemias. Bacteraemias have been recorded in subjects who have received attenuated *Salmonella* vaccines containing only *aro* deletions.

S. typhi (Ty2 *aro*C^{-} *ssa*V^{-}) ZH9 is derived from the virulent *S. typhi* strain Ty2. This strain has been used as a background strain for constructing candidate typhoid vaccines evaluated in volunteers previously. The currently USA licensed live *Salmonella* vaccine is derived from this strain.

2.3 Non-Clinical Summary

The attenuation of *S. typhi* (Ty2 *aro*C^{-} *ssa*V^{-}) ZH9 has been demonstrated directly by comparing replication in human macrophage-like cells of *S. typhi* (Ty2 *aro*C^{-} *ssa*V^{-}) ZH9 with *S. typhi* (Ty2 *aro*C^{-}) DTY8, which harbours a single *aro*C mutation, in the presence and absence of aromatic compound supplements.

Replication of *S. typhi* (Ty2 *aro*C^{-}) DTY8 strain in U937 macrophage-like cells was much lower in the absence of additional aromatic amino acids (1 round of replication within macrophages in a 48 hour period compared to 4 to 5 rounds when aromatic amino acids were added). Thus the *aro*C mutation reduces replication of *S. typhi* in macrophages, in agreement with a previous study (Lowe *et al.*, 1999).

In contrast, *S. typhi* (Ty2 *aro*C^{-} *ssa*V^{-}) ZH9, showed no increase in cell numbers, even in the presence of aromatic compound supplements, over the 48-hour period. The *ssa*V mutation therefore completely prevents replication in human macrophage like cells for 48 hours. These results confirm the previous studies, demonstrating the role of SPI-2 genes in bacterial proliferation within macrophages (Ochman *et al.*, 1996) and demonstrate the utility of mutations in SPI-2 as an attenuation strategy for *Salmonella*. Emergent Product Development UK Ltd considers this to be a key safety study.

S. typhimurium strains harbouring either a single *aro*C deletion mutation or deletion mutations in both *aro*C and *ssa*V have been used to demonstrate the additional utility of the *ssa*V mutation over that of the *aro*C mutation in immuno-compromised mice.

Experiments in IFNγ knock out mice demonstrated that SPI-2 mutants of *Salmonella,* including a strain carrying exactly the same mutation as the candidate vaccine strain *S. typhi* (Ty2 *aro*C^{-} *ssa*V^{-}) ZH9 were unable to kill immuno-compromised animals whereas attenuated strains containing only *aro* mutants did kill immuno-compromised mice. The combination of an SPI-2 mutation such as *ssa*V with an *aro* mutation in a *Salmonella* vaccine candidate therefore provides additional safety assurance for use of these strains in humans.

A single dose study designed to assess the potential toxicity of *S. typhi* (Ty2 *aro*C^{-} *ssa*V^{-}) ZH9 when administered to CD-1 mice by oral gavage at doses of 5 x 10^7 and 5 x 10^9 colony forming units (CFU) showed no systemic toxicological changes. There were no significant changes in bodyweight, food consumption, haematology, clinical chemistry, organ weights, macroscopic or microscopic pathology in the 15-day period following dosing. Recovery of *S. typhi* (Ty2 *aro*C^{-} *ssa*V^{-}) ZH9 was demonstrated in mouse faeces in both treated groups from 1 to 5 days after dosing.

2.4 Clinical Summary

Summary of Clinical Data to Date

To date *S. typhi* (Ty2 *aro*C⁻ *ssa*V⁻) ZH9 has been administered to over 100 healthy adult volunteers in four studies. In the first study (Study MS01.01) 9 subjects (3 per cohort) received doses of either, 10^7, 10^8 or 10^9 CFU of a frozen formulation of the vaccine. In the second study (Study MS01.03) 48 subjects (16 per cohort) received doses of 5 x 10^7, 5 x 10^8 or 5 x 10^9 CFU of a freeze-dried formulation of the *S. typhi* (Ty2 *aro*C⁻ *ssa*V⁻) ZH9 vaccine. In the third study (Study MS01.04) 32 subjects received one dose of 5 x 10^9 CFU of the freeze-dried formulation and in the fourth study (MS01.07) a further 16 subjects received one dose of 5 x 10^9 CFU of the freeze-dried formulation. These studies indicate that the vaccine is immunogenic with a good safety profile.

Summary of Data from Study MS01.01

Study MS01.01 was an open non-controlled, in-patient dose escalating exploratory study conducted under a Doctors and Dentists exemption certificate (DDX), designed to determine the safety and tolerability of three dose levels of the attenuated live *Salmonella typhi* strain *S. typhi* (Ty2 *aro*C⁻ *ssa*V⁻) ZH9 (Hindle *et al*, 2002). Nine (3 cohorts of 3) healthy adult volunteers received a single oral dose of 10^7, 10^8 or 10^9 CFU of the vaccine strain.

This study demonstrated that the candidate, live oral typhoid vaccine strain *S. typhi* (Ty2 *aro*C⁻ *ssa*V⁻) ZH9 was well tolerated. There was no evidence of systemic spread of the bacteria. All blood and urine cultures remained negative in all subjects at all time-points examined. Persistent shedding (defined as shedding beyond Day 7) in the stools was not observed in any of volunteers of the three dose groups. The adverse events reported by single subjects that were potentially related to study medication were abdominal discomfort, headache, diarrhoea (more than 3 unformed stools in a 24-hour period), nausea and vomiting. *S. typhi* (Ty2 *aro*C⁻ *ssa*V⁻) ZH9 was determined to be immunogenic at doses of 10^7 to 10^9 CFU in human subjects.

Summary of Data from Study MS01.03

Study MS01.03 was a single dose, placebo controlled, double blind, dose-escalating study conducted under an Investigational New Drug (IND). The study was designed to determine the safety, tolerability and immunogenicity of three dose levels of M01ZH09 which consisted of a freeze dried formulation of the live attenuated vaccine strain *S. typhi* (Ty2 *aro*C⁻ *ssa*V⁻) ZH9 and excipients. A total of 60 healthy adult volunteers were recruited in 3 cohorts of 20. In each cohort, 16 subjects were randomised to receive vaccine and 4 to receive placebo. The cohorts were dosed sequentially receiving doses of 5x10^7 CFU of *S. typhi* (Ty2 *aro*C⁻ *ssa*V⁻) ZH9, 5x10^8 CFU of *S. typhi* (Ty2 *aro*C⁻ *ssa*V⁻) ZH9, 5x10^9 CFU of *S. typhi* (Ty2 *aro*C⁻ *ssa*V⁻) ZH9 or placebo. Dose escalation proceeded only after a safety review group had determined that it was appropriate to escalate the dose.

Safety Data (MS01.03)

None of the blood cultures taken during the study was positive for *S. typhi* (Ty2 *aro*C⁻ *ssa*V⁻) ZH9. Faecal shedding of *S. typhi* (Ty2 *aro*C⁻ *ssa*V⁻) ZH9 did not continue beyond 6 days for any subject, at any of the dose levels. The number of subjects with faecal shedding of the vaccine strain increased with increasing dose level.

There were no serious adverse events (SAEs) related to study medication. A total of three SAEs were reported, two in the 28-day period following dosing, both involved minor surgical procedures for pre-existing conditions. The third SAE was reported in the six month follow up period when a subject was admitted to hospital for adjustment of medication for a pre-existing condition.

Forty-six subjects reported adverse events affecting the gastrointestinal system including flatulence, abdominal pain or discomfort and nausea. A total of 41 subjects reported adverse events of the nervous system; with the most frequently reported being headache. Thirty-six subjects reported general disorders with fatigue being the commonest event in this category. Thirty subjects reported adverse events related to the musculoskeletal system with musculoskeletal pain and myalgia being the commonest complaints. The majority of adverse events were of mild severity although some subjects reported moderate abdominal pain; these were spread evenly across all dose groups. Adverse events that were reported as severe included headaches, which were reported by subjects in all groups that received the vaccine, infections (urinary tract and ear infections neither caused by the vaccine strain), hunger, and nasal congestion. Eight subjects reported pyrexia (temperatures of 37.4 – 38.7°C), with no evidence of a bacteraemia. One case was not considered related to study medication. Of the remaining 7 cases, 2 subjects were in the placebo group 2 were in the 5 x 10^8 and 3 in the 5 x 10^9 CFU group. Four of these events were of mild severity and were considered possibly related to study medication. One subject in the 5 x 10^9 CFU dose group reported a body temperature of 38.3°C approximately 16 hours after receiving the dose of vaccine, the event was considered as severe and probably related to study medication. A second subject in the 5 x 10^9 CFU group, had a swinging temperature up to 38.7 °C on study Days 10 and 11. Ten subjects reported rigors/chills, 1 in the placebo group, 2 in the 5 x 10^7 CFU dose group, 4 in the 5 x 10^8 CFU dose group and 3 in the 5 x 10^9 CFU dose group. Three of these subjects reported having pyrexia concurrent with their rigors/chills. Only one of the rigors/chills was reported as being severe, 10 days after dosing with 5 x 10^9 CFU, and this subject also experienced a pyrexia. There was no statistically significant difference in the incidence of adverse events between subjects receiving the vaccine and those who received placebo and M01ZH09 was well tolerated by all of the subjects dosed.

Immunogenicity (MS01.03)

All three dose levels were shown to be immunogenic, with the highest dose being immunogenic in all subjects. In both the 5 x 10^7 and 5 x 10^8 CFU dose groups 9 (56%) subjects in each group mounted an IgA immune response on Day 7 or an IgG response on Day 28. This number increased to 16 (100%) in the 5 x 10^9 CFU dose group. None of the subjects that received placebo mounted an immunological response against *S. typhi* (Ty2 *aro*C$^-$ *ssa*V$^-$) ZH9.

Summary of Data from Study MS01.04

Study MS01.04 was a single centre, open, randomised, parallel group study conducted under an IND. The study was designed to determine the immunogenicity and safety of two different presentations of 5 x 10^9 CFU of M01ZH09 administered as a single oral dose to healthy volunteers. A total of 32 healthy adult volunteers were recruited to the study. Sixteen subjects were randomised to receive vaccine presentation 1 and 16 to vaccine presentation 2.

Subjects receiving vaccine presentation 1 consumed 100 mL of 2% (w/v) sodium bicarbonate solution between 5 and 20 minutes prior to receiving the vaccine administered in a further 50 mL of 2% (w/v) sodium bicarbonate solution. The solution was prepared using bottled water.

Subjects randomised to vaccine presentation 2, received the vaccine in 150 mL of a solution containing 1.75% (w/v) sodium bicarbonate, 1.1% (w/v) ascorbic acid and 0.02% (w/v) aspartame. The solution was prepared using tap water.

All 32 subjects who entered the study received their dose of vaccine and 31 subjects completed the study. One subject withdrew their consent and therefore did not complete the study.

Safety Data (MS01.04)

The majority of subjects (84%) in the study reported at least one post-vaccination adverse event. A total of 15 (94%) of subjects that received M01ZH09 in presentation 1 reported 44 events. In comparison a total of 12 (75%) subjects that received M01ZH09 in presentation 2, reported 51 events. The majority of the events were gastrointestinal or nervous system disorders.

Gastrointestinal disorders were reported by 8 (50%) subjects in each presentation group. The commonest events specific to the gastrointestinal system were loose stools, which were reported by 4 and 2 subjects for presentations 1 and 2 respectively and abdominal pain, which were reported by 3 and 1 subject(s) respectively. Nervous system disorders were reported by 6 (38%) subjects in presentation 1 and by 10 (63%) subjects in presentation 2. The commonest event specific to the nervous system was headache, which was reported by 6 and 9 subjects for presentations 1 and 2 respectively. Respiratory, thoracic and mediastinal disorders were reported by 3 (19%) subjects receiving presentation 1 and 5 (31%) subjects receiving presentation 2.

There were no deaths or SAEs reported. The majority of events were of mild or moderate severity with only 7 of the 95 events being reported as severe.

There was little difference between the presentations in terms of the occurrence of adverse events.

Immunogenicity Data (MS01.04)

The majority of subjects showed an immune response following administration of M01ZH09. Fifteen subjects (94%) who received presentation 1 mounted an immune response as demonstrated by an increase in the number of antibody secreting cells (ASCs) to 4 or more and /or seroconversion of the subjects with the presence of IgG antibody specific to *S. typhi* LPS. Fourteen subjects (93%) who received presentation 2 mounted an immune response. Both groups had a statistically significant difference to an assumed response rate of 50%, ($p \leq 0.001$; 95% CIs (69.8%, 99.8%) for presentation 1 and (68.1%, 99.8%) for presentation 2).

For the IgG response, the proportion of subjects who had an immune response was higher for presentation 1 (13 subjects, 81%, 95% CI (54.4%, 96.0%)) than for presentation 2 (8 subjects, 53%, 95% CI (26.6%, 78.7%)).

For the IgA response, 14 subjects in both groups mounted an immune response but the proportion of subjects who had an immune response was lower for presentation 1 (14 subjects, 88%, 95% CI (61.7%, 98.4%)) than for presentation 2 (14 subjects, 93%, 95% CI (68.1%, 99.8%)) as one subject in presentation 2 did not attend the Day 7 visit to provide a blood sample.

There was no evidence of a difference between the groups in the proportion of subjects mounting either an IGA or an IGG immune response (p=1.0; 95% CI (-16.9%, 17.7%)).

Similar results were seen for the IgA response alone (p=1.0; 95% CI (-26.4%, 14.7%)).

For the IgG response there were differences in the proportions of subjects showing an immune response although this difference was not statistically significant (p=0.1351; 95% CI (-3.8%, 59.6%)).

Overall the immunogenicity was comparable for both presentations and the safety profiles were similar. Based on these data it seems appropriate to use presentation 2 in MS01.07 as it is prepared and administered in a more convenient way and also the total amount of bicarbonate consumed is lower (2.6g compared to 3g).

Summary of Data from Study MS01.07

Study MS01.07 was a single centre, placebo-controlled, single-blind, randomised, parallel group, single oral dose study. The study was designed to determine the immunogenicity and safety of 5 x 10^9 CFU of M01ZH09 administered as a single oral dose to healthy adult volunteers of Vietnamese origin. The study planned to recruit 28 subjects. A total of 27 healthy adult volunteers were actually recruited to the study. It was anticipated that 16 subjects would be randomised to receive vaccine and 12 subjects to receive placebo. The actual number of subjects that received vaccine was 16 and the number that received placebo was 11.

Subjects received either vaccine or placebo in 150 mL of a buffer solution containing 1.75% (w/v) sodium bicarbonate, 1.1% (w/v) ascorbic acid and 0.02% (w/v) aspartame. The buffer solution was prepared using drinking water.

All 27 subjects that entered the study received their dose of either vaccine or placebo and all 27 completed the study.

Safety Data (MS01.07)

Nine (82%) of the subjects who received placebo reported a total of 14 adverse events and 8 (50%) of subjects that received M01ZH09 reported 23 adverse events. The only events reported by 10% or more of subjects were diarrhoea, abdominal pain and headache. There were no apparent differences between the placebo and active groups in the frequencies of these events.

None of the subjects recorded a bacteraemia, a serious or a severe adverse event and none of the subjects withdrew due to an adverse event. The majority of events were of mild severity with only 5 events considered to be of moderate severity. None of the adverse events reported required treatment or medication for the adverse event.

None of the subjects recorded or reported a fever or had any clinically significant changes in laboratory parameters.

None of the subjects experienced persistent faecal shedding of *S. typhi* (Ty2 *aro*C$^-$ *ssa*V$^-$) ZH9. (Persistent faecal shedding was defined as shedding of *S. typhi* (Ty2 *aro*C$^-$ *ssa*V$^-$) ZH9 beyond day 7).

Overall M01ZH09 appears to have been very well tolerated and to have a good safety profile in healthy adult Vietnamese subjects.

Immunogenicity Data (MS01.07)

Sera from blood were taken fortnightly until Day 28 and will be obtained 6 month post vaccination for the evaluation of IgG responses against *S. typhi* LPS, *S.typhi* flagellin and *S.typhi* Vi as assessed by the ELISA technique. Peripheral blood mononuclear cells (PBMC) were isolated from blood

collected on Days 0 and 7 and assayed, using the ELISPOT technique, for the presence of ASCs secreting IgA specific for *S.typhi* LPS.

Subjects were considered to have had an immune response if they achieved a Day 7 result of = 4 ASCs per 10^6 PBMC, secreting IgA specific for *S. typhi* LPS detected by ELISPOT assay, (assuming a Day 0 result of <4 ASCs per 10^6 PBMC), and/or undergo seroconversion for serum IgG to LPS. Seroconversion for serum IgG to LPS is defined as a four-fold or greater increase in the serum IgG antibody on serial dilution by ELISA, to *S. typhi* LPS between Day 0 and Day 28.

None of the subjects that received the placebo mounted an immunological response against *S.typhi* (Ty2 *aro*C¯ *ssa*V¯) ZH9. 11/15 subjects (73%) within the group receiving a single dose (5 x 10^9 CFU) of M01ZH09 typhoid vaccine demonstrated an LPS specific IgA response between day 0 and day 7. 3/16 subjects (19%) demonstrated an LPS specific IgG response between day 0 and day 28.

Overall 69% (11/16) of subjects within the group receiving a single dose (5 x 10^9 CFU) of *S.typhi* (Ty2 *aro*C¯ *ssa*V¯) ZH9 satisfied the primary immunogenicity endpoint of the MS01.07 study (ITT population).

2.5 Rationale for Dose

The purpose of this study is to investigate the safety and immunogenicity of the vaccine in a different age group to that tested to date. To achieve this it is essential to use a dose that is known to be immunogenic. In study MS01.03 the maximum dose administered (5 x 10^9 CFU) was immunogenic in all 16 subjects dosed. The lower doses investigated in Study MS01.03 were immunogenic in approximately 50% of the subjects. The safety profiles were similar for all groups; hence, at the doses investigated toxicity did not limit dosing. In Study MS01.04, the dose studied (5 x 10^9 CFU) was immunogenic in 29 of 32 subjects. In Study MS01.07, the dose studied (5 x 10^9 CFU) was immunogenic in 11 of 16 Vietnamese subjects. There is precedent for using the same dosage of live typhoid vaccine in children as adults. School children in the Vivotif (Ty21a) clinical trial in Chile received the same regimen as used for the licensed product in adults (Ferreccio et al., 1989). Moreover, Vivotif is licensed for use in the US; it is indicated for immunisation of adults and children greater then 6 years of age using the same immunisation schedule. In line with other live vaccines, such as Stamaril (yellow fever) and Meruvax II (rubella), the same parenteral dose is given to children as it is to adults. For these reasons it has been determined that the appropriate dose for this study is a nominal dose of 5 x 10^9 CFU of *S. typhi* (Ty2 *aro*C⁻ *ssa*V⁻) ZH9.

2.6 Study Population

The study will recruit 150 healthy paediatric Vietnamese subjects. Subjects will be of either gender and will be between 5 and 14 years of age, who are willing to take part, whose parents or guardians give written permission and who meet all the inclusion and exclusion criteria.

At least 70% of the subjects in each group will be aged 5 to 10 years inclusive.

2.7 Risks and Benefits

The anticipated risks of the study are in line with the side effect profile seen to date with M01ZH09. The most likely adverse effects are flatulence, abdominal cramps/discomfort, loose/unformed

stools, headache, nausea, vomiting, chills/rigors, minor elevations in body temperature (up to 38°C) and mild muscle and joint pain in subjects receiving the active vaccine.

For subjects receiving the active vaccine there is a theoretical risk of developing a mild typhoid-like condition. Theoretically there is also a potential risk of subjects developing a clinically significant bacteraemia, or becoming a carrier of the *Salmonella* strain *S. typhi* (Ty2 *aro*C^{-} *ssa*V^{-}) ZH9, and while these outcomes are considered highly unlikely and have not been seen to date in the populations studied, both these conditions would require treatment with antibiotics (refer to Section 6.6 for recommended antibiotic treatment).

There is a potential risk of subjects developing allergic symptoms, such as rashes or anaphylaxis. There may also be other as yet unforeseen risks.

The investigational product will be administered in a bicarbonate solution containing 1.75% (w/v) sodium bicarbonate, 1.1% (w/v) ascorbic acid and 0.02% (w/v) aspartame. Subjects aged 5 to 10 years will receive the vaccine or placebo in 75 mL of bicarbonate solution. Subjects aged 11 to 14 years will receive the vaccine or placebo in 150 mL of bicarbonate solution. The solution is prepared from effervescent bicarbonate tablets using drinking water. The bicarbonate solution may induce some abdominal discomfort and/or cramping around the time of dosing.

Subjects who have received 5 x 10^9 CFU of M01ZH09 have developed an immune response. At present it is not known if the immune response generated by M01ZH09 is protective against infection from *S. typhi*. There can be no assumed benefit for the subjects receiving M01ZH09 even if an immune response is detected; such a response cannot be assumed to be protective. If any protection is obtained, the duration of any such protection is currently unknown.

2.8 Study Treatment

Subjects will receive a single dose of M01ZH09 administered orally. The oral route of administration is the normal route of entry for *S. typhi* hence it is appropriate to administer the vaccine by the same route. There will be 2 groups of subjects; 100 subjects will be randomised to receive a nominal dose of 5 x 10^9 CFU of M01ZH09 oral vaccine and 50 subjects will receive placebo. M01ZH09 is supplied as a freeze-dried formulation of the vaccine strain, plus excipients, in single dose vials. The placebo is supplied as a freeze-dried formulation of vaccine excipients, in single dose vials.

It is planned that a total of 100 subjects will receive the M01ZH09 vaccine in a bicarbonate solution containing 1.75% (w/v) sodium bicarbonate, 1.1% (w/v) ascorbic acid and 0.02% (w/v) aspartame. The solution is prepared from bicarbonate tablets using drinking water.

It is planned that a total of 50 subjects will receive placebo, consisting of the vaccine excipients in a bicarbonate solution containing 1.75% (w/v) sodium bicarbonate, 1.1% (w/v) ascorbic acid and 0.02% (w/v) aspartame. The solution is prepared from bicarbonate tablets using drinking water.

Subjects aged 5 to 10 years will receive the vaccine or placebo in 75 mL of bicarbonate solution. Subjects aged 11 to 14 years will receive the vaccine or placebo in 150 mL of bicarbonate solution

2.9 Compliance Statement

This study is to be performed in compliance with this protocol, local and national guidelines and regulations and the following code of federal regulations;

- 21CFR 50 subpart B – Informed Consent of Human Subjects
- 21CFR54 – Financial Disclosure by Clinical Investigators
- 21CFR312.60 – General Responsibilities of Investigators
- 45CFR46 – Protection of Human Subjects
- Current ICH Guideline for Good Clinical Practice

Quality assurance systems and procedures will be implemented to assure the quality of every aspect of the study.

3 STUDY PURPOSE, OBJECTIVES AND ENDPOINTS

3.1 Purpose

The purpose of the study is to determine the safety and immunogenicity of the oral vaccine M01ZH09 in healthy paediatric Vietnamese subjects. The clinical studies performed to date with M01ZH09 have assessed safety, tolerability and immunogenicity in adult subjects in the USA, UK and Viet Nam. Later in the clinical development of M01ZH09, Phase III field trials will be necessary in an area where typhoid fever is endemic. Potential areas for such a study exist in Viet Nam and Nepal. Field trials of a typhoid vaccine will involve children, as children under 10 years of age make up a significant proportion of the population that experience typhoid disease. The purpose of this study is to demonstrate immunogenicity and safety in a paediatric Vietnamese population prior to initiating field trials where it is anticipated that the youngest subjects that will be vaccinated will be 5 years of age.

3.2 Objective

To evaluate the safety and immunogenicity of M01ZH09 typhoid vaccine (oral live *S. typhi* (Ty2 *aro*C^{-} *ssa*V^{-}) ZH9) at a nominal dose of 5 x 10^{9} CFU in healthy paediatric subjects from Viet Nam.

3.3 Safety Endpoints

3.3.1 Primary

The proportion of subjects reporting SAEs attributed to the study medication.

3.3.2 Secondary

The proportion of subjects;

- Experiencing an elevated body temperature, of 38.5°C or greater, in the 14 days following dosing, attributed to study medication.
- Demonstrating persistent (after Day 7) faecal shedding of *S. typhi* (Ty2 *aro*C^{-} *ssa*V^{-}) ZH9.
- Withdrawn from the study due to adverse events, including bacteraemia, attributed to study medication.
- With clinically significant changes in laboratory parameters, from Day 0 to any time post dosing, which are attributed to study medication.

3.4 Immunogenicity Endpoints

Assessment of the immune response will be made by determining the proportion of subjects who;

- Develop a positive immune response* to *S. typhi* LPS as assessed by an increase in *S. typhi* LPS specific IgG at either Day 14 or Day 28.
- Develop a positive immune response* to *S. typhi* LPS as assessed by an increase *S. typhi* LPS specific IgA at either Day 7 or Day 14.

- At Day 7, have = 4 ASCs per 10^6 PBMC, secreting IgA specific for *S. typhi* LPS detected by ELISPOT assay

*The definition of a ‘positive immune response’ will be determined following the completion of assay development.

4 STUDY DESIGN

4.1 Summary of Study Design

This is a placebo-controlled, parallel group, randomised, single-blind, single centre study. Subjects will be randomly allocated to one of two treatment groups and will receive either a single dose of M01ZH09 at a nominal dose of 5 x 10^9 CFU or placebo. A total of 100 subjects will take M01ZH09 and 50 will take placebo.

4.2 Rationale for Study Design

The study will be conducted following the principles of a double blind study, as per the accepted norm as the gold standard for these types of study. While this is the intent, perfect blinding cannot be guaranteed for a number of reasons (see Section 4.5, Blinding). The placebo serves as a measure of background adverse events and wild-type typhi immunogenicity markers. Allowing more children in the range 5-10 years of age will increase the data available in the age group to likely be employed for Phase III field trials.

4.3 Study Population

The study will recruit 150 healthy paediatric Vietnamese subjects. Subjects will be of either gender and will be between 5 and 14 years of age, inclusive, who are willing to take part, whose parents or guardians give written permission and who meet all the inclusion and exclusion criteria.

At least 70% of the subjects in each group will be aged 5 to 10 years inclusive.

4.4 Rationale for Dose

The purpose of this study is to investigate the safety and immunogenicity of the vaccine in a different age group to that tested to date. To achieve this it is essential to use a dose that is known to be immunogenic. In previous studies, the maximum dose administered (5 x 10^9 CFU) has been shown to be immunogenic. Lower doses investigated in previous studies were immunogenic in approximately 50% of the subjects. The safety profiles have been similar for all doses administered; hence, at the doses investigated, toxicity did not limit dosing. There is precedent for using the same dosage of live typhoid vaccine in children as adults. School children in the Vivotif (Ty21a) clinical trial in Chile received the same regimen as used for the licensed product in adults (Ferreccio et al., 1989). Moreover, Vivotif is licensed for use in the USA; it is indicated for immunisation of adults and children greater than 6 years of age using the same immunisation schedule. In line with other live vaccines, such as Stamaril (yellow fever) and Meruvax II (rubella), the same parenteral dose is given to children as it is to adults. For these reasons it has been determined that the appropriate dose for this study is a nominal dose of 5 x 10^9 CFU of *S. typhi* (Ty2 *aro*C$^-$ *ssa*V$^-$) ZH9.

4.5 Blinding

Active vaccine and matching placebo will be packaged identically but with a unique sequential number. This number will enable identification of the contents in case of emergency when used

with the randomisation code break. Preparation of both active and placebo doses will be identical. Hence the investigator and his staff will be essentially blinded in that knowledge of the product supplied to the subjects will remain unknown. However, three areas remain where unintentional unblinding may occur.

1. The placebo and M01ZH09 solutions differ slightly in taste and aroma. Individual subjects will be unaware of any differences in dosing solutions and consequently will be blinded to their treatment in the study. The investigator may detect a difference between the products based on the aroma. A double observer design would reduce this risk but personnel with paediatric expertise at the site are limited, and a double observer design is unlikely to be possible.
2. Subjects will have stool samples taken for culturing and it is anticipated that subjects receiving M01ZH09 will shed *S. typhi* (Ty2 *aro*C^{-} *ssa*V^{-}) ZH9 in their stools. Results from the stool microbiology will therefore potentially unblind the investigator. It is intended to request that the Investigator does not review these microbiology reports for at least the first 14 days post dosing.
3. Subjects will have blood samples taken for the determination of IgG and IgA specific to *S. typhi*. Subjects that develop an immune response will clearly be identifiable as having received M01ZH09. For this reason it is intended to request that the Investigator does not review these immunology results for at least the first 14 days post dosing.

4.6 Study Treatments

The vaccine and placebo will be supplied in labelled single dose glass vials, in labelled boxes, to the pharmacy at the study site. Each vial will contain a nominal dose of 5 x 10^9 CFU *S. typhi* (Ty2 *aro*C^{-} *ssa*V^{-}) ZH9 or placebo. M01ZH09 is supplied as a freeze-dried formulation of the vaccine strain, plus excipients, in single dose vials (see Section 6.1). The placebo contains only the excipients. Subjects will receive one single oral dose of 5 x 10^9 CFU *S. typhi* (Ty2 *aro*C^{-} *ssa*V^{-}) ZH9 or placebo.

Effervescent bicarbonate tablets containing 2.6 g sodium bicarbonate, 1.65 g ascorbic acid and 30 mg aspartame will be supplied. The bicarbonate tablets will be dissolved in drinking water. Once the tablet has completely dissolved, the vaccine will be added to this solution.

The pharmacist must confirm receipt of study medication in writing. Emergent Product Development UK Ltd or its authorised agent will retain samples of study medication.

4.7 Duration of Subject Participation

Each subject's participation in the study will be for up to 28 days during the screening period plus 28 days following the administration of the investigational medicinal product (IMP), making a total of 56 days. Recruitment of 150 subjects at the study site is expected to take approximately six months.

4.8 Stopping Rules

4.8.1 Study as a Whole

Emergent Product Development UK Ltd or the Investigator may decide to stop the study or part of the study at any time. If the study is prematurely terminated or suspended, the Investigator should promptly inform the subjects and ensure appropriate therapy and follow-up. Furthermore, the Investigator should promptly inform the Ethics Committee and provide a detailed written explanation. The pertinent regulatory authorities must be informed according to national regulations.

Should there be any serious AEs which are both unexpected and at least possibly related to the IMP following vaccination, there will be a suspension of dosing, a full review of the safety data and the study may be stopped.

If, following dosing with IMP, subjects experience signs and symptoms as discussed below (See Table 1), dosing of further subjects will be suspended until a full review of the safety and laboratory data has been completed by the Investigator and the sponsor's Medical Monitor. The continuation of dosing will be dependent upon the findings of the review.

Table 1: Rules for suspension of dosing

	**Events that would trigger a suspension of dosing**		
	Moderate	Severe	
**Gastrointestinal symptoms***	30% of, or 6 subjects, dosed, whichever is the greater number of subjects, with moderate vomiting or diarrhoea.	10% of, or 2 subjects, dosed, whichever is the greater number of subjects, with severe nausea, vomiting or diarrhoea.	
**Systemic reactions**	30% of, or 6 subjects, dosed, whichever is the greater number of subjects, with moderate fever (= 38.5°C).	10% of, or 2 subjects, dosed, whichever is the greater number of subjects with severe fever (= 39.5°C).	1 subject with a positive blood culture for a *S. typhi* strain.
**Laboratory values****	30% of, or 6 subjects, dosed, whichever is the greater number of subjects with moderate abnormalities.	10% of, or 2 subjects, dosed, whichever is the greater number of subjects with severe abnormalities.	
**Other Adverse Reactions**		10% of, or 2 subjects, dosed, whichever is the greater number of subjects with a related possibly or probably (Section 8.1.4)) SAE.	

* See Table 2

** See Table 3

Table 2: Definition of severity for gastrointestinal symptoms

Symptom	Moderate	Severe
**Nausea**	Affects the subject's normal daily activities.	Prevents the subject's normal daily activities or requires confinement to bed.
**Vomiting***	More than 4 episodes in a 24-hour period but no evidence of clinically significant dehydration.	More than 6 episodes in a 24-hour period or evidence of significant dehydration as a result of the vomiting.
**Diarrhoea***	More than 4 unformed stools in a 24-hour period but no evidence of clinically significant dehydration.	More than 6 unformed stools in a 24-hour period or evidence of significant dehydration as a result of the diarrhoea.

* If the number of episodes are not reached but hospital admission is required, the event will still be classified as severe, as well as classifying as a Serious Adverse Event.

Table 3: Definition of severity for laboratory value abnormalities

Laboratory test	Moderate	Severe
**WBC increase**	= 15 to 20 x 10^9/L	> 20 x 10^9/L
**WBC decrease**	< 2.5 to 1.5 x 10^9/L	< 1.5 x 10^9/L
**Liver function tests (AST and ALT)**	2.5 to 3.5 x ULN*	> 3.5 x ULN*

* ULN = Upper Limit of Normal

In the event of a *salmonella* bacteraemia, dosing will be suspended until it has been determined whether the strain responsible for the bacteraemia is *S. typhi* (Ty2 *aro*C$^-$ *ssa*V$^-$) ZH9. If it is determined that the bacteraemia should be attributed to *S. typhi* (Ty2 *aro*C$^-$ *ssa*V$^-$) ZH9 or the vaccine strain cannot be ruled out as the causative agent, dosing will be suspended and an SAE report will be provided to the FDA and other relevant regulatory authorities.

Other signs and symptoms may also lead to suspension of dosing or other changes in the intended protocol pending review of the safety data and culture results.

4.8.2 Individual Subjects

If a subject wishes to leave the study at any time, or if either of the subject's parents or guardians withdraw their permission, the subject will be permitted to leave the study without any penalty. The collection of safety data is important for the safety of the individual subject and therefore, subjects who withdraw will be encouraged to monitor their temperature and adverse events and return for safety follow-up and the collection of samples for reasons of safety. It will be stressed to the subject and his/her parents or guardians that if the subject becomes unwell they should, at a minimum, contact the investigative site and preferably visit the investigative site to ensure the vaccine has not caused any adverse events.

4.9 Randomisation Codes and Breaking the Code

Subjects will be allocated a 'Screening Number' on entry into the study (signature of the written permission document). This number, together with their initials will be used to identify them until they are deemed eligible to receive study treatment at Visit 1.

At Visit 1, all subjects eligible to receive study treatment will be allocated a unique subject number (this number will be recorded in the Visit 1CRF together with the Screening Number; at subsequent visits, the subject will be identified by this number and their initials only). This number will be allocated strictly sequentially (within each age-group cohort) in accordance with the randomisation schedule. The randomisation schedule will determine if a subject receives vaccine or placebo. The vials of IMP will be provided to the study site pre labelled with subject numbers.

Code break will be provided to the Investigator. The code for a particular subject can be broken in a medical emergency if knowing the identity of the treatment allocation would influence the treatment of the subject. Whenever a code is broken, the person breaking the code must record the time, date and reason as well as their initials in the source documents.

If the site needs to break the code, the sponsor should, if possible, be contacted prior to breaking the code. In all cases, the Study Monitor must be notified within 24 hours after the code has been broken.

All code break (whether broken or not) must be kept throughout the study period. Codes will be checked for integrity and collected by the Study Monitor at study site closure.

5 SELECTION AND WITHDRAWAL OF SUBJECTS

5.1 Inclusion Criteria

Subjects must satisfy the following criteria

1. Healthy paediatric subjects aged 5 to 14 years inclusive, of Vietnamese origin, who are able and willing to take part in the trial and whose parents or guardians give written permission for their child's participation, following a detailed explanation of the study.
2. Subjects who will be available for the duration of the study and available for scheduled and potential additional visits.

5.2 Exclusion Criteria

Subjects meeting any of the following criteria must be excluded from the study

1. Subjects with any clinically significant medical or psychiatric condition or clinically significant abnormal serum biochemistry or haematology results that, in the opinion of the Investigator, preclude participation in the study.
2. Subjects whose body weight is under 17kg (5-10 year olds), or under 27kg (11-14 year olds)
3. Female subjects who are pregnant (confirmed with urinary pregnancy test) or breast-feeding, or of childbearing potential and unwilling to use a reliable method of contraception (oral contraceptives, barrier method with spermicidal preparation or abstinence) throughout the study period.
4. Subjects who have a known hypersensitivity to two or more of the following antibiotics; ciprofloxacin, azithromycin or trimethoprim-sulfamethoxazole or have used antibiotics/antibacterials within 14 days prior to administration of study medication.
5. Subjects who have a known hypersensitivity to any component of the vaccine or bicarbonate solution used in this study, including subjects with phenylketonuria or those who have experienced anaphylactic shock after any vaccination.
6. Subjects who received Vivotif, either as a licensed or investigational product, in the last 10 years or any other vaccine against *S. typhi*, whether licensed or investigational, in the last 5 years, or who have ever suffered from typhoid fever.
7. Subjects with direct contact with patients in special care units or immuno-compromised individuals.
8. Subjects who have a positive bacterial culture of their faecal sample, obtained at the screening visit, for any *Salmonella* species.
9. Subjects with a known impairment of immune function including acquired immune deficiency syndrome or those receiving (or having received in the 6 months prior to screening) cytotoxic drugs, immunosuppressive therapy (including systemic corticosteroids).
10. Subjects who are HIV positive or have family members who are HIV positive. ('Family members' includes immediate and extended family living in the same residential unit, or relations with whom the subject has regular and frequent contact).

11. Subjects with a significant acute febrile illness (body temperature of 38.0°C or more) at time of dosing.
12. Subjects who have chronic diseases: Chronic diseases will include all autoimmune and immuno-compromising conditions and any other chronic condition which, at the judgement of the Investigator, may put the subject at higher risk of side effects from the study vaccine. Conditions in the latter category might include unexplained anaemia, hepato-biliary disease, uncontrolled hypertension, subjects with prosthetic joints or heart valves, etc.
13. Subjects with a current problem, based on history, of substance abuse or with a history of substance abuse that, in the opinion of the investigator, might interfere with participation in the study.
14. Subjects who are currently involved in a clinical study, have taken an investigational drug or have received investigational or licensed vaccines in the 4 weeks before screening, or anticipate receiving a vaccine, other than study medication, during the first 4 weeks, post vaccination, of the study.

Subjects excluded for medical reasons will be referred to appropriate medical care services or counselling.

5.3 Withdrawal Criteria

Emergent Product Development UK Ltd or the Investigator may decide to stop the study or part of the study at any time. If a study is prematurely terminated or suspended, the Investigator should promptly inform the subjects and ensure appropriate therapy and follow-up. Furthermore, the Investigator should promptly inform the Ethics Committee and provide a detailed written explanation. The pertinent regulatory authorities must be informed according to the national regulations.

A subject may be withdrawn from the study at the discretion of the Investigator or the Sponsor, if judged non-compliant with study procedures or due to safety concerns.

A subject must be withdrawn if any of the following apply:

- The wish of the subject or either of his/her parents or guardians
- Intolerable adverse event(s)
- Clinically significant abnormal laboratory findings (which in the opinion of the Investigator and/or Sponsor precludes further participation in the study)
- Non-compliance, (subject does not attend a minimum of 3 visits post dosing)
- Use of treatments not permitted during the study (see section 6.6)
- Investigator's decision that withdrawal from further participation would be in the subject's best interest.
- Pregnancy. Any subject who becomes pregnant will be followed up to determine the outcome of the pregnancy. The Investigator will be required to inform Emergent Product Development UK Ltd of a subject's pregnancy and the estimated date of delivery. Emergent Product

Development UK Ltd will follow up with the Investigator around the estimated time of delivery to determine the outcome of the pregnancy.

This is a single dose study; therefore subjects withdrawing after dosing would not be scheduled to receive any additional medication. Blood draws for immunogenicity assessments would be discontinued, although safety assessments would be continued. The collection of safety data is critically important and therefore subjects will be encouraged to return for the collection of samples for safety, whether they have been withdrawn for reasons of safety, have withdrawn their assent or have had their parental permission withdrawn. As many safety samples as possible will be collected.

5.4 Subject Replacement

There will be no replacement of subjects who have received study medication. Subjects who have permission and assent forms signed but withdraw before receipt of study medication will be replaced.

5.5 Follow-up of Withdrawn Subjects

If a subject withdraws from the study before the final visit then, if possible, all the Day 28 assessments will be completed. If the subject cannot attend for a final assessment then as a minimum, the End of Study Form must be completed. If a subject withdraws due to an adverse event then the subject must be followed until the event ends or stabilises.

6 TREATMENT OF SUBJECTS

6.1 Study Products

Subjects will receive a single oral dose of vaccine or placebo. The oral route of administration is the normal route of entry for *S. typhi* hence it is appropriate to administer the vaccine by the same route. M01ZH09 is supplied as a freeze-dried formulation of the vaccine strain, plus excipients, in single dose vials. The excipients are shown in Table 4 below;

Table 4: Excipients included in the single dose vials

There will be two groups; 100 subjects will be randomised to receive a nominal dose of 5×10^9 CFU of M01ZH09 and 50 subjects will be randomised to receive placebo.

Group 1. Active, consisting of 5×10^9 CFU of M01ZH09 vaccine strain *S. typhi* (Ty2 *aro*C^- *ssa*V^-) ZH9 administered in a bicarbonate solution.

Group 2. Placebo, consisting of the vaccine excipients administered in a bicarbonate solution.

The bicarbonate solution is made from an effervescent bicarbonate tablet containing 2.6 g sodium bicarbonate, 1.65 g ascorbic acid and 30 mg aspartame. Each bicarbonate tablet will be dissolved in 150 mL of drinking water. The vaccine or placebo will be dissolved in the appropriate volume of this solution containing 1.75% (w/v) sodium bicarbonate, 1.1% (w/v) ascorbic acid and 0.02% (w/v) aspartame.

Subjects aged 5 to 10 years will receive the vaccine or placebo in 75 mL of bicarbonate solution. Subjects aged 11 to 14 years will receive the vaccine or placebo in 150 mL of bicarbonate solution.

6.2 Method of Administration of Each Dose

The method for the reconstitution and administration of the vaccine will be detailed in a separate document provided to the Pharmacist and Investigator.

Subjects will fast for at least 2 hours prior to taking the dose of medication and for 90 minutes after dosing (nothing to eat or drink, with the exception of water). The subject will remain at the Investigative site, under observation, for at least 90 minutes following dosing.

6.3 Packaging and Labelling of Study Products

Vials of IMP and bicarbonate tablets will be provided to the study site in cartons. One carton will contain one vial and one bicarbonate tablet.

Each glass vial will be labelled with the following information as a minimum:

Study Number: MS01.08
Subject Number:
M01ZH09 oral typhoid vaccine or placebo
Caution: New Drug - Limited by Law to investigational use
FOR ORAL ADMINISTRATION ONLY
Keep refrigerated at 2°C to 8°C. DO NOT FREEZE
Packaging reference number:
Emergent Product Development UK Ltd, Berkshire RG41 5TU, UK

Each tablet will be labelled with the following information as a minimum:
Study Number: MS01.08
Subject Number:
Bicarbonate tablet for live attenuated vaccine (contains 2.6 g sodium bicarbonate, 1.65 g ascorbic acid and 30 mg aspartame)
Caution: New Drug - Limited by Law to investigational use
For oral use dissolved in water in accordance with the pharmacy instructions.
Not to be swallowed directly.
Store between 2°C and 27°C
Lot number:
Packaging reference number:
Emergent Product Development UK Ltd, Berkshire RG41 5TU, UK

The cartons will be sealed with tamper evident seals. The labels will contain as a minimum:
Study Number: MS01.08
Subject Number
M01ZH09 oral typhoid vaccine or placebo, plus bicarbonate tablet
Caution: New Drug - Limited by Law to investigational use
FOR ORAL ADMINISTRATION ONLY
Keep refrigerated at 2°C to 8°C. DO NOT FREEZE
Lot / Packaging reference number:
Sponsor: Emergent Product Development UK Ltd, Berkshire RG41 5TU, UK
Investigator: Dr Hien, Hospital for Tropical Diseases, Ho Chi Minh City, Viet Nam

6.4 Storage and Drug Accountability of Study Products

Vials of study medication must be kept refrigerated at 2°C to 8°C.

The responsible pharmacist will maintain a Medication Accountability Log. This will include the date, description and quantity of study medication received at the study site, as well as a record of when and to whom it was dispensed.

All unused supplies will be retained at site until Emergent Product Development UK Ltd gives instructions for their return or disposal.

No study products may be dispensed to any person not enrolled in the study or used for purposes other than those described in this protocol.

6.5 Other Materials

Emergent Product Development UK Ltd will provide syringes for use during reconstitution of the vaccine, and dosing containers.

6.6 Concomitant Medication

The use of all medication including ‘over the counter’ and herbal products taken in the 4 weeks prior to screening and during the course of the study, up to Day 28, must be detailed in the case report form (CRF). Subjects are to try to avoid all antibiotic/antibacterial use for 14 days before and after dosing. Should a subject require antibiotics he/she must inform the Investigator and may be withdrawn from the study depending on the timing in relation to the vaccination or have their data excluded from the PP analysis. Subjects must not receive any vaccine other than M01ZH09 during the four weeks, post dose, of the study.

Oral contraception is allowed under the protocol. However, should a female subject using oral contraception develop diarrhoea or require antibiotic therapy she will be requested to use additional barrier methods of contraception in combination with a spermicidal preparation.

If a subject has

- a single positive blood culture, or
- a positive stool culture for *S. typhi* at any time after Day 7

he/she must be treated with antibiotics. The first line antibiotic will be ciprofloxacin 20 mg/kg twice daily, taken orally for 10 days. The second line antibiotic will be azithromycin. The dose of azithromycin is dependent upon the age of the subject. Subjects aged 5 to 7 years should receive 5 mL (200 mg) orally once daily for 7 days, subjects aged 8 to 11 years should receive 7.5 mL (300 mg) orally once daily for 7 days and subjects 11 to 14 years should receive 10 mL (400 mg) orally once daily for 7 days. The third line antibiotic is trimethoprim 40 mg/sulfamethoxazole 200 mg per 5 mL of suspension twice daily, 10 mL taken orally for 10 days. In all cases antibiotic therapy will be initiated as soon as the culture results are known.

Any subject who returns for unscheduled visits after Day 14 and has positive blood or stool cultures for *S. typhi* will be treated as a potential carrier and treated with antibiotics and followed up until the infection is clear. Antibiotics will be administered as outlined above at the discretion of the Investigator. The duration of treatment may be up to 28 days to ensure the infection is clear.

The vaccine strain has been shown to be sensitive to ciprofloxacin, azithromycin and trimethoprim/sulphamethoxazole. Ciprofloxacin has been shown to be the treatment of choice for chronic carriers of wild type *S. typhi*.

Anti-pyretic medications (for example, aspirin, ibuprofen, naproxen sodium) should not be taken for minor aches/pains or injuries as such medication may mask a fever. Paracetamol is an

appropriate concomitant medication for these purposes. Should a subject experience a fever (body temperature = 38.5°C) it is important that he/she contact the investigative site and visit to be assessed. Subjects or their parents/guardians should not initiate anti-pyretic medication unless instructed to do so by the Investigator. Anti-pyretics may be administered under the direction of the Investigator for the management of a diagnosed fever.

6.7 Compliance

The study staff will administer the study medication and observe the subjects' compliance.

6.8 Visits and Procedures

6.8.1 Screening period (Day –28 to Day –1)

The parents or guardians of all subjects will provide written permission before any study related procedure is carried out. Where appropriate, with regard to age and understanding, the subject will be asked to sign an assent form indicating their willingness to take part in the study. After written permission has been obtained, the subject will be allocated a 'Screening Number' which will be used, together with his/her initials as their unique identifier until dosing at Visit 1. The following will be recorded/performed:

- Demographic data
- Medical history, including any allergies, and vaccination history
- Prior concomitant medications (in previous 4 weeks before screening), particularly antibiotic use and including herbal and over the counter medications
- Physical examination including height, weight and vital signs (blood pressure, temperature and pulse) and an examination for any signs of splenomegaly.
- Draw blood for:
    - Serum biochemistry and haematology (Section 7.2.1)
    - HIV test
- Obtain urine sample for:
    - Urine pregnancy test (females of 11 years and older only)
    - Dipstick test, and if the dipstick is positive for leucocyte esterase and nitrites, a separate sample will be sent for microscopy, culture and sensitivity
- Obtain stool sample for culture for *Salmonella spp.*
- Make an appointment for suitable subjects to attend for Visit 1 (randomisation and administration of the study medication) within the next 4 weeks. Instruct subject to bring a stool sample to the next visit.

6.8.2 Visit 1: Day of Dosing/ Day 0

This visit should take place within 28 days of the screening visit, following receipt of the full laboratory results. After the laboratory results have been reviewed, those subjects who continue to meet the inclusion criteria, and do not meet any of the criteria for exclusion, will continue in the study. The following tasks will be performed:

- Collect stool sample subject has brought to clinic. (Note: This sample will not be cultured prior to dosing but if it is subsequently shown to be positive for any Salmonella strain the data from the subject will be excluded from the PP analysis).
- Review the inclusion and exclusion criteria for continued study eligibility
- Review medical history and record any changes since screening
- Enquire about and record any adverse events since the last visit (see Section 6.8.9) to include an examination for any signs of splenomegaly
- Enquire about and record any changes in concomitant medication since screening

If the subject has received antibiotic therapy within 14 days of this visit, arrange for the subject to return for dosing if this is possible within 28 days of the screening visit. If not, please withdraw the subject from the study.

Subjects who are withdrawn at Visit 1, under these circumstances and cannot be dosed within 28 days of the Screening Visit may be re-screened. Under these circumstances the subject must be re-consented.

If the subject remains eligible:

- Determine and record vital signs (blood pressure, temperature and pulse)

If oral temperature is 38°C or more, arrange for the subject to return for dosing if this is possible within 28 days of the screening visit. If not, please withdraw the subject from the study.

Subjects who are withdrawn at Visit 1, under these circumstances and cannot be dosed within 28 days of the Screening Visit may be re-screened. Under these circumstances the subject must be re-consented.

- Obtain urine sample for:
    - Urine pregnancy test (females aged 11 years and older only)
    - Dipstick test, and if the dipstick is positive for leucocyte esterase and nitrites, a separate sample will be sent for microscopy, culture and sensitivity
- If the subject remains eligible, allocate the next available age-group specific Subject Number to the subject and enter this into the Visit 1 header of the CRF and the associated medication pack bearing this 'Subject Number'.
- Draw blood for:
    - Serum biochemistry and haematology
    - Serum IgG (ELISA assay)

- Serum IgA (ELISA assay)
- Determining the number of ASC secreting IgA (ELISPOT assay; only for subjects aged at least 11 years and weighing at least 27 kg)

- After ensuring that the subject has fasted for at least 2 hours, administer the dose of study medication (if the subject vomits within 1 hour of administration then he/she will need to receive a repeat dose).
- Record pulse and blood pressure at 30, 60 and 90 minutes after dosing
- Subjects will remain at the study site for at least 90 minutes following administration of study medication so that any adverse events may be recorded.
- Subjects must not eat or drink (with the exception of water) for 90 minutes following vaccination
- Issue a digital thermometer for recording body temperature orally. Subjects and their parents/guardians will be instructed to take body temperature readings each day in the morning when they get up and in the evening when they go to bed and to record the measurement in the Diary Card.
- Issue a Diary Card and instruct the subject and their parents/guardians on its completion (Section 7.2.4)
- Make an appointment for Visit 2/ Day 1 and instruct the subject to bring a stool sample to the visit.

6.8.3 Visit 2: Day 1

The subject will return to the clinic approximately 24 hours after dosing, where the following assessments will be completed:

- Collect the stool sample that the subject has brought to the clinic for culture of vaccine strain *S. typhi* (Ty2 *aro*C^{-} *ssa*V^{-}) ZH9
- Determine and record vital signs (blood pressure, temperature and pulse)
- Review Diary Cards and summarise data in CRF
- Enquire about and record any adverse events since the last visit (see Section 6.8.9) to include an examination for any signs of splenomegaly
- Enquire about and record any changes in concomitant medication since the last visit
- Make an appointment for Study Day 2 and instruct the subject to bring a stool sample to the visit

6.8.4 Visits 3, 4, 5, 6 and 7: Days 2, 3, 4, 5 and 6

The subject will return to the clinic where the following assessments will be completed:

- Collect the stool sample that the subject has brought to clinic for culture of vaccine strain *S. typhi* (Ty2 *aro*C^{-} *ssa*V^{-}) ZH9

- Determine and record vital signs (blood pressure, temperature and pulse)
- Review Diary Cards and summarise data in CRF
- Enquire about and record any adverse events since the last visit (see Section 6.8.9) to include an examination for any signs of splenomegaly
- Enquire about and record any changes in concomitant medication since the last visit
- Make an appointment for a clinic visit on the following day and instruct the subject to bring a stool sample to the visit

6.8.5 Visit 8: Day 7

The subject will return to the clinic 7 days after vaccination, where the following assessments will be completed:

- Collect the stool sample that the subject has brought to clinic for culture of vaccine strain *S. typhi* (Ty2 *aro*C^{-} *ssa*V^{-}) ZH9
- Determine and record vital signs (blood pressure, temperature and pulse)
- Review Diary Cards and summarise data in CRF.
- Enquire about and record any adverse events since the last visit (see Section 6.8.9) to include an examination for any signs of splenomegaly
- Enquire about and record any changes in concomitant medication since the last visit
- Draw blood for:
    - Serum biochemistry and haematology
    - Serum IgA (ELISA assay)
    - Determining the number of ASC secreting IgA (ELISPOT assay; only for subjects aged at least 11 years and weighing at least 27 kg)
- Obtain urine sample for:
    - Dipstick test, and if the dipstick is positive for leucocyte esterase and nitrites, a separate sample will be sent for microscopy, culture and sensitivity and also to determine if vaccine strain *S. typhi* (Ty2 *aro*C^{-} *ssa*V^{-}) ZH9 is present
- Make an appointment for Study Day 8 and instruct the subject to bring a stool sample to the visit

6.8.6 Visit 9, 10, 11, 12, 13 and 14: Days 8, 9, 10, 11, 12 and 13

The subject will return to the clinic where the following assessments will be completed:

- Collect the stool sample that the subject has brought to clinic for culture of vaccine strain *S. typhi* (Ty2 *aro*C^{-} *ssa*V^{-}) ZH9
- Determine and record vital signs (blood pressure, temperature and pulse)
- Review Diary Cards and summarise data in CRF.

- Enquire about and record any adverse events since the last visit (see Section 6.8.9) to include an examination for any signs of splenomegaly
- Enquire about and record any changes in concomitant medication since the last visit
- Make an appointment for a clinic visit on the following day and instruct the subject to bring a stool sample to the visit

6.8.7 Visit 15: Day 14

The subject will return to the clinic 14 days after dosing, when the following assessments will be completed:

- Collect the stool sample that the subject has brought to clinic for culture of vaccine strain *S. typhi* (Ty2 *aro*C^- *ssa*V^-) ZH9
- Determine and record vital signs (blood pressure, temperature and pulse)
- Review Diary Cards and summarise data in CRF
- Enquire about and record any adverse events since the last visit (see Section 6.8.9) to include an examination for any signs of splenomegaly
- Enquire about and record any changes in concomitant medication since the last visit
- Draw blood for:
    - Serum biochemistry and haematology
    - Serum IgG (ELISA assay)
    - Serum IgA (ELISA assay)
- Obtain urine sample for:
    - Dipstick test, and if the dipstick is positive for leucocyte esterase and nitrites, a separate sample will be sent for microscopy, culture and sensitivity, and also to determine if vaccine strain *S. typhi* (Ty2 *aro*C^- *ssa*V^-) ZH9 is present
- Make an appointment for Study Day 28.

6.8.8 Visit 16: Day 28

The subject will return to the clinic 28 days after dosing, where the following assessments will be completed:

- Physical examination including vital signs (blood pressure, temperature and pulse)
- Enquire about and record any adverse events since the last visit (see Section 6.8.9) to include an examination for any signs of splenomegaly
- Enquire about and record any changes in concomitant medication since the last visit
- Draw blood for:
    - Serum biochemistry and haematology
    - Serum IgG (ELISA assay)

- Obtain urine sample for:
    - Pregnancy test (females of 11 years and older only)
    - Dipstick test, and if the dipstick is positive for leucocyte esterase and nitrites, a separate sample will be sent for microscopy, culture and sensitivity, and also to determine if vaccine strain *S. typhi* (Ty2 *aro*C⁻ *ssa*V⁻) ZH9 is present

6.8.9 Specific Safety Reviews at Each Visit

Visits to the clinic are scheduled daily, for the first 14 days post-dosing. At these visits, the study staff will obtain and record the subject's blood pressure, oral body temperature, pulse and assess for signs of splenomegaly. The Investigator will enquire of the subject, and the parents or guardians, as to whether the subject has had any signs or symptoms of typhoid fever. Specifically, enquiries will relate to whether the subject has had any of the following;

- Temperature of 38.5°C or above.
- Flushed face
- Hot, dry skin
- Low output of urine and/or dark urine
- Loss of interest in eating
- Constipation or diarrhoea
- Vomiting
- Headache
- Aching all over
- Nausea
- Chills
- Abdominal pains, cramps or discomfort

6.8.10 Unscheduled Visits

In addition to the scheduled visits described above, the subjects' oral body temperature will be taken at home, twice daily. If a subject shows sustained signs and symptoms of a clinical infection defined as a confirmed fever of = 38.5°C, or other signs and symptoms as outlined above, they will be instructed to make additional unscheduled visits to the clinic. At such visits the subject will be assessed and biological samples taken for culture if clinically indicated. If a clear diagnosis can be made, as in the case of Dengue fever, the subject will be managed accordingly. Should a confirmed fever of = 39.0°C be recorded twice over a 48-hour period, or a severe fever of = 39.5°C ever be recorded, blood sample for cultures will be taken to investigate if *S. typhi* (Ty2 *aro*C⁻ *ssa*V⁻) ZH9 is present in the blood.

7 IMMUNOGENICITY, SAFETY AND LABORATORY ASSESSMENTS

7.1 Assessments of Immunogenicity

Full details of the immunogenicity assays including sample collection, processing, storage and analysis are provided in the Laboratory Manual. ELISPOT assays will be conducted at the study centre, ELISA samples for assay will be shipped to Emergent Product Development UK Ltd after preparation and freezing, under the supervision of Dr Jeremy Farrar the Principal Investigator for this study and in accordance with the instructions provided by Emergent Product Development UK Ltd.

7.1.1 Measure of Antibody Secreting Cells by ELISPOT

ELISPOT assay of the number of ASCs secreting IgA to LPS will be conducted on blood samples taken on Days 0 and 7 for subjects aged at least 11 years and weighing at least 27kg. The assay will be undertaken within 24 hours of the sample being taken.

Human PBMCs will be separated from whole blood by density gradient centrifugation. After centrifugation the lymphocytes will be collected and washed. The cells will then be suspended in culture medium and different dilutions added to microtitre wells coated with *S. typhi* LPS. Following overnight incubation, the cells will be removed. Zones of antigen-specific secreted IgA antibody bound to the plate will be visualised by the addition of alkaline phosphatase-labelled anti-human IgA antibodies followed by the addition of 5-bromo-4-chloro-3-indolyl-phosphate (BCIP)/Nitro Blue Tetrazolium (NBT) substrate. Regions of specific antibody appear as blue ELISPOTS, and these will be observed and counted using an inverted microscope and then photographed. The number of ASC/10^6 cells can then be calculated from the results obtained with different dilutions of PBMCs.

7.1.2 Analysis of LPS Specific Immunoglobulin G by the ELISA Assay

Serum for ELISA assays for IgG will be frozen immediately after collection on Days 0, 14 and 28, and shipped to Emergent Product Development UK Ltd for analysis after all subjects have completed 28 days for LPS-specific IgG serum antibody.

Microtitre plates are coated with *S. typhi* LPS and blocked with a non-specific protein to mask unbound binding sites. Human serum samples are added to the wells and any antibodies present that are specific for the *S. typhi* LPS will bind to the coated plate. These bound antibodies are then detected using a double antibody system. First, an unlabelled anti-human IgG antibody is added which, in turn, is detected by a horseradish peroxidase (HRP)-labelled conjugate which causes a detectable colour change when 3,3,5,5, Tetramethylbenzidine (TMB) substrate is added. The products are quantified with an automated ELISA reader at 450 nm and the concentration of anti-LPS human IgG is determined by extrapolation of the dilution that is within the range of the standard curve.

7.1.3 Analysis of LPS Specific Immunoglobulin A by the ELISA Assay

Serum for ELISA assays for IgA will be frozen immediately after collection on Days 0, 7 and 14 and shipped to Emergent Product Development UK Ltd for analysis after all subjects have completed 14 days for LPS-specific IgA serum antibody.

Microtitre plates are coated with *S. typhi* LPS and blocked with a non-specific protein to mask unbound binding sites. Human serum samples are added to the wells and any antibodies present that are specific for the *S. typhi* LPS will bind to the coated plate. These bound antibodies are then detected using a double antibody system. First, an unlabelled anti-human IgA antibody is added which, in turn, is detected by a horseradish peroxidase (HRP)-labelled conjugate which causes a detectable colour change when 3,3,5,5, Tetramethylbenzidine (TMB) substrate is added. The products are quantified with an automated ELISA reader at 450 nm and the concentration of anti-LPS human IgA is determined by extrapolation of the dilution that is within the range of the standard curve.

7.2 Assessment for Safety

7.2.1 Laboratory Tests

Subjects will be asked to attend the clinic every day for the first 14 days of the study. At these visits the Investigator (a paediatrician) will obtain and record the subject's blood pressure, oral body temperature, pulse and assess for signs of splenomegaly. The Investigator will enquire of the subject, and the parents or guardians, as to whether the subject has had any signs or symptoms of typhoid fever. Specifically, enquiries will relate to whether the subject has had any of the following;

- Temperature of 38.5°C or above
- Flushed face
- Hot, dry skin
- Low output of urine and/or dark urine
- Loss of interest in eating
- Constipation or diarrhoea
- Vomiting
- Headache
- Aching all over
- Nausea
- Chills
- Abdominal pains, cramps or discomfort

Based on the examination and the responses to the questioning, the Investigator will initiate appropriate investigations, including biological samples for culture, to determine if the child has an infection and the cause of the infection.

The faecal shedding pattern of the bacterial strain has been determined in adults with no subject, to date, shedding the vaccine strain for more than 7 days after dosing. The faecal shedding pattern in children is expected to be the same but this study will investigate whether paediatric subjects shed for a shorter or longer period than adults.

The following tests will be performed on blood samples collected at screening and Days 0, 7, 14 and 28:

Biochemistry:

Sodium, potassium, chloride, creatinine, blood urea, aspartate aminotransferase (AST), alanine aminotransferase (ALT), alkaline phosphatase, total bilirubin, gamma-glutamyl transferase (gGT), serum amylase, total protein and albumin.

Haematology:
Haemoglobin, haematocrit, red blood cell count, white blood cell count (total and differential), platelet count.

In total, approximately 27.5 mL of blood will be taken from each subject, **aged 5 to 10 years** and weighing more than 17 kg, for the immunogenicity and safety tests during the 28-day phase of the study. The maximum amount taken will be no more than 5.5mL at any one visit.

In total, approximately 43.5 mL of blood will be taken from each subject, **aged 11-14 years** and weighing more than 27 kg, for the immunogenicity and safety tests during the 28-day phase of the study. . The maximum amount of blood taken will be no more than 13.5 mL at any one visit.

If a subject shows sustained signs and symptoms of a clinical infection, defined as a confirmed fever of = 38.5°C, they will be instructed to make additional unscheduled visits to the clinic. At such visits the subject will be assessed and biological samples taken for culture if clinically indicated. Should a confirmed fever of = 39.0°C be recorded twice over a 48-hour period, or a severe fever of = 39.5°C ever be recorded, blood cultures will be taken to investigate if *S. typhi* (Ty2 *aro*C^{-} *ssa*V^{-}) ZH9 is present in the blood. Each blood culture would require a blood draw of 1-3 mL.

Urine samples will be tested, by dipstick, at screening and Days 0, 7, 14 and 28 for the presence of nitrites and leucocyte esterase. If the dipstick is positive for nitrites and leucocyte esterase a sample will be sent for culture and sensitivity.

Stool samples will be collected for culture at all visits except Day 28. Details of the assay method will be documented in the Laboratory Manual.

Any post vaccination unformed/loose stool experienced by the subject will be recorded as an adverse event.

7.2.2 Physical Examination

A standard physical examination will be performed at the Screening visit and at Day 28. Any abnormalities will be described in the CRF. This will include assessments of the following:

- Cardiovascular system
- Respiratory system
- Nervous system

- Skin and appendages
- Abdominal/gastrointestinal system
- Musculoskeletal system
- Ear, nose and throat

7.2.3 Vital Signs

Vital signs will be determined and recorded at each study visit and recorded on the CRF. Vital signs include:

- Sitting (5 minute) systolic and diastolic blood pressure
- Oral temperature (see also section 7.2.4)
- Pulse rate

7.2.4 Diary Card

Each subject will complete a Diary Card daily for the 14 days following administration of study medication. The subject will record his/her temperature or have their temperature recorded, twice in the morning (15 minutes apart) soon after getting up and twice at bedtime (15 minutes apart), as well as recording all adverse events. Subjects will be instructed that:

- Should their temperature be confirmed as = 38.5°C on both readings, measured 15 minutes apart, or reach 39.0°C or above on any reading, to contact and visit the investigative site as soon as is practical, to have a clinical assessment and initiation of appropriate investigations and therapy.
- Should they feel feverish they should make additional recordings of their temperature and contact the investigative site
- Should they experience any of the following events with moderate or severe intensity they should contact the investigative site and be prepared to make an additional visit:
    - Flushed face
    - Hot, dry skin
    - Low output of urine and/or dark urine
    - Loss of interest in eating
    - Constipation or diarrhoea
    - Vomiting
    - Headache
    - Aching all over
    - Nausea
    - Chills
    - Abdominal pain, cramps or discomfort

- Subjects will also conduct a stool assessment after each bowel movement. The time of passing the stool will be recorded and the stool assessed as ‘Normal’, ‘Soft but in one piece’, ‘thick liquid’, or ‘with rice and water’. The observer will also be asked to record if any blood was visible.

8 ADVERSE EVENTS

8.1 Definitions

8.1.1 Adverse Event

An adverse event (AE) is any untoward medical occurrence in a subject administered a pharmaceutical product and which does not necessarily have to have a causal relationship with this treatment.

An AE can therefore be any unfavourable and unintended sign (including a clinically significant abnormal laboratory finding*, for example), symptom, or disease temporally associated with the use of a medicinal product, whether or not considered related to the medicinal product. This includes events from the first study related activity after the subject and his/her guardian has signed the informed permission and assent forms. The following should not be recorded as AEs, if recorded at screening:

- Pre-planned procedure, unless the condition for which the procedure was planned has worsened from the first study related activity after the subject and his/her guardian has signed the informed permission and assent forms.
- Pre-existing conditions found as a result of screening procedures (unless they get worse)

***** A ‘clinically significant abnormal laboratory’ finding is defined as any clinical laboratory abnormality that suggests a disease and/or organ toxicity and is of a severity which requires active management (i.e. more frequent follow-up or diagnostic investigation) or is clinically significantly outside of the normal range.

8.1.2 Serious Adverse Event

A serious adverse event (SAE) is an experience that, at any dose, results in any of the following outcomes:

- Death
- A life-threatening* experience
- In-patient hospitalisation or prolongation of existing hospitalisation
- A persistent or significant disability/ incapacity
- A congenital anomaly/ birth defect
- Important medical events that may not result in death, be life-threatening* or require hospitalisation may be considered a SAE when, based upon appropriate medical judgement, they jeopardise the subject and may require medical or surgical intervention to prevent one of the outcomes listed in this definition.

* The term life-threatening in the definition of SAE refers to an event in which the subject was at risk of death at the time of the event. It does not refer to an event that hypothetically might have cause death if it was more severe.

Bacteraemia should also be reported to Akos Ltd following the SAE reporting procedure. If the blood culture grows the M01ZH09 vaccine strain or if the cause of the bacteraemia is undetermined Akos Ltd will report the event as a serious, unexpected, related, adverse event.

8.1.3 Severity Assessment Definitions

The Investigator will be asked to evaluate the severity of the AE according to the following scale:

- Mild: Transient or mild discomfort (<48 hours); no medical intervention/therapy required.
- Moderate: Mild to moderate limitation in activity - some assistance may be needed; none or minimal medical intervention/therapy required.
- Severe: Marked limitation in activity, some assistance usually required; medical intervention/therapy required, hospitalisation possible.

8.1.4 Relationship to Study Product Assessment Definitions

The Investigator will be requested to evaluate the relationship between the AE and the study medication according to the following scale:

- Probably: Good reason and sufficient documentation to assume a causal relationship
- Possibly: A causal relationship is conceivable and cannot be dismissed
- Unlikely: The event is most likely related to an aetiology other than the investigational product

8.1.5 Outcome Categories and Definitions

The outcome will be evaluated according to the following scale:

- Recovered: Fully recovered or by medical or surgical treatment the condition has returned to the level observed from the first study related activity after the subject signed the informed permission and assent.
- Stabilised: This outcome should only be used for cancer events and chronic conditions that cannot be normalised by medical or surgical treatment. This term should only be used when the subject has completed the protocol
- Recovered with sequelae
- Not yet recovered
- Fatal
- Unknown

8.1.6 Fever Definition

Fever is defined as an oral temperature of = 38.5ºC. Severe fever is defined as an oral temperature of = 39.5ºC. All subjects will be asked to take their temperatures twice a day in the morning when they get up and in the evening when they go to bed for the first 14 days post-dosing.

8.2 Collection, Recording and Reporting of Adverse Events

All AEs must be recorded in the subject's CRF and must be monitored until there is either a return to normal or until the condition becomes stable or is resolved.

All SAEs, whether or not they are judged to be related to the study drug, should be reported, by the Investigator, to Akos Ltd, upon learning of the SAE. SAEs must be reported within 24 hours of knowing about the event or, ideally, within 24 hours of the occurrence of the event. The Investigator must complete the Akos Ltd SAE form with as much information as is available at the time of completion and fax the form immediately to the number provided.

Akos Ltd, the Study Monitor, the Medical Monitor, or a representative of Emergent Product Development UK Ltd, may follow up by requesting clarification or additional information on the SAE, from the Investigator.

It is the responsibility of the Sponsor to report serious, unexpected adverse drug reactions (ADR)s to the relevant regulatory authorities and other Investigators as required under ICH, GCP and relevant regulatory reporting requirements.

It is the responsibility of the Investigator (with the assistance of Emergent Product Development UK Ltd) to report any serious, unexpected ADRs to the Ethics Committee in accordance with ICH guidelines and local requirements.

8.3 Follow-up of Adverse Events

Events discovered or spontaneously reported to the Investigator within 28 days of taking the medication will be documented in the CRF. All SAEs must be followed until all queries related to these AEs have been resolved.

All other AEs must be followed until the Day 28 visit.

The Investigator must forward follow-up information on SAEs to Akos Ltd within 5 days of obtaining the follow-up information. Only changed or new information should be supplied.

8.4 Safety Committee

A safety committee will not be required for this study.

8.5 Pregnancy

Pregnancy is not an AE but the outcome may be. Study subjects must notify the Investigator immediately if they become pregnant. Subjects who become pregnant will be followed up to determine the outcome of the pregnancy. The Investigator will be required to inform Emergent Product Development UK Ltd of a subject's pregnancy and the estimated date of delivery.

Emergent Product Development UK Ltd will follow up with the Investigator around the estimated time of delivery to determine the outcome of the pregnancy.

8.6 Overdose

Overdose is unlikely, as subjects will receive only one dose of study medication and under the supervision of research staff. If a subject accidentally receives more than one dose of study medication, the subject will be observed and treated with antibiotics only if they shed beyond 7 days, develop clinical signs and symptoms of a bacteraemia, develop a persistent silent bacteraemia or become a chronic carrier.

9 STATISTICS

9.1 Sample Size Calculation

The planned number of subjects is 150 in total, 100 should receive M01ZH09 oral typhoid vaccine and 50 placebo. No formal statistical calculations of sample size were appropriate. The number to include was based upon clinical judgement for both the assessment of safety and determination of immunogenicity. The latter from a consideration of the immunogenicity rates observed in M01ZH09 adult studies completed thus far and the immunogenicity rates observed in children, for typhoid vaccines currently on the market.

9.2 Statistical Methods

9.2.1 General Points

Analyses will be primarily conducted to estimate parameters of interest such as the proportion of subjects that develop an immune response. Due to the exploratory nature of the study, no adjustment will be made for multiplicity. All available data from withdrawn subjects will be included in the analysis up to the time of withdrawal where possible.

Any deviation(s) from the original statistical analysis plan (SAP) (which will be finalised prior to database lock) will be described and justified in the final report.

9.2.2 Admission Profile

Demographics and other baseline data will be listed and summarised. Continuous variables (such as age, weight) will be summarised by numbers of subjects, means, medians, standard deviations, minima and maxima. Categorical variables (such as race, sex) will be summarised by counts and percentages in each category.

9.2.3 Study Populations

The safety population will include all subjects who received a dose of study medication.

The intention-to-treat (ITT) population will include all subjects who received a dose of study medication who have post-baseline immunogenicity data available. The ITT population will be considered the primary population of interest for the immunogenicity endpoints.

The per-protocol (PP) population analysis will be a subset of the ITT population and will be subjects who are not major protocol violators with regards to the inclusion/exclusion criteria, are compliant with study medication and concomitant medications. The SAP will define the exact criteria for exclusion from the PP population.

9.2.4 Safety Endpoints

9.2.4.1 Primary

The proportion of subjects reporting SAEs attributed to the study medication

9.2.4.2 Secondary

The proportion of subjects;

- Experiencing symptomatic elevated body temperature, of 38.5 C or greater, in the 14 days following dosing, attributed to study medication.
- Demonstrating persistent (after Day 7) faecal shedding of *S. typhi* (Ty2 *aro*C$^-$ *ssa*V$^-$) ZH9.
- Withdrawn from the study due to adverse events, including bacteraemia attributed to study medication
- With clinically significant changes in laboratory parameters, from Day 0 to any time post dosing, which are attributed to study medication.

9.2.5 Immunogenicity Endpoints

Assessment of the immune response will be made by determining the proportion of subjects who;

- Develop a positive immune response* to *S. typhi* LPS as assessed by an increase in *S. typhi* LPS specific IgG at either Day 14 or Day 28.
- Develop a positive immune response* to *S. typhi* LPS as assessed by an increase in *S. typhi* LPS specific IgA at either Day 7 or Day 14.
- At Day 7, have = 4 ASCs per 10^6 PBMC, secreting IgA specific for *S. typhi* LPS detected by ELISPOT assay**.

*The definition of a 'positive immune response' will be determined following the completion of assay development.

**An ELISPOT Day 0 result of = 4 ASCs per 10^6 PBMC constitutes a pre-vaccination positive and the subject will be excluded from the analysis of the ELISPOT results.

9.3 Methods of Analysis for Safety Variables

All safety summaries will be carried out on the safety population defined in Section 9.2.3.

Summary statistics will be used to present the number of subjects receiving M01ZHZ09 oral typhoid vaccine and placebo.

The numbers of subjects reporting adverse events will be summarised by body system and by MedDRA (version 8.1 or later) preferred term. All adverse experiences recorded after vaccination will be summarised and listed. Adverse events with missing or incomplete onset dates will be assumed to be post-dosing adverse events. Summaries will also be produced showing adverse events by severity, seriousness and relationship to treatment.

The numbers of subjects withdrawn from the study due to adverse events, including bacteraemia attributed to *S. typhi* (Ty2 *aro*C$^-$ *ssa*V$^-$) ZH9, will be presented separately.

Counts will be based on the number of subjects experiencing an event and not the number of events experienced e.g. if a subject reports the same adverse event on three occasions that adverse event will be counted once. Subjects reporting more than one adverse event in a body system will be counted only once in the body system total.

The number and percentage of subjects will be presented, by Visit and overall, that:

- Experience symptomatic elevated body temperature in the 14 days following dosing, attributed to study medication
- Demonstrate persistent (after Day 7) faecal shedding of *S. typhi* (Ty2 *aro*C^{-} *ssa*V^{-}) ZH9
- Have clinically significant changes in laboratory parameters from Day 0 to any time post dosing which are attributed to study medication.

Laboratory results will be listed with values that are outside the laboratory normal range flagged. For each parameter the overall numbers of values that are within the pre-determined clinically important range at Day 0 but outside the range post-treatment will be summarised.

Vital signs and physical examination results will be summarised by visit.

9.4 Methods of Analysis for Immunogenicity Endpoint

The primary outcome measure in this study will be the proportion of subjects with a positive immune response (a positive serum IgG and/or a positive serum IgA response and/or an increase in the number of ELISPOTS). The proportion of subjects who satisfy the immunogenicity endpoint will be compared between the vaccine and placebo groups. The treatment difference and associated 95% confidence interval will be presented.

The number and percentage of subjects with an immune response will be presented along with the two-sided 95% confidence interval for each treatment group.

In addition, the number and percentage of subjects with defined immune response will be presented by type of assay.

10 DIRECT ACCESS TO SOURCE DATA/ DOCUMENTS

This study is undertaken on the express understanding that the Investigator/Institution will permit study-related monitoring, audits, Ethics Committee review and regulatory inspection(s), providing direct access to source data/documents.

Direct access includes permission to examine, analyse, verify and copy any records and reports that are important to evaluation of the clinical study.

Prior to study start, a source data verification (SDV) document will be created and agreed with the Investigator. This document will specify the source documents for the key items of data collected during the study. SDV by the Study Monitor will be conducted in accordance with this SDV document. Source data such as laboratory results will be signed and dated by the Investigator to confirm that these have been medically reviewed.

11 QUALITY CONTROL AND QUALITY ASSURANCE

Data will be recorded on CRFs provided by Emergent Product Development UK Ltd. Study specific instructions on the completion of CRFs will be given to the Investigator and appointed staff.

By signing the Investigator Statement at the end of the CRF, the Investigator confirms that the information provided by the Investigator and his appointed staff is complete and correct.

Correction to CRFs prior to collection will be made by drawing a straight line through the incorrect data and by writing the correct value next to data that has been crossed out. Each correction must be initialled, dated and explained (if necessary) by the Investigator or the Investigator's authorised staff.

Correction to CRFs after collection from the Investigator's site must be documented on a Query Resolution Form.

Source data such as laboratory results will be signed and dated by the assessor.

During the course of the study, the Study Monitor will visit the investigational site at intervals appropriate to recruitment and volume and quantity of data. The purpose of these visits is to ensure that the CRFs are completed correctly, the protocol is adhered to, IMP is properly accounted for and to collect completed pages of the CRFs.

The Monitor will review source documents directly, e.g. subjects' notes, to ensure correct transcription and quality of data.

Study files and data may be audited by an independent auditor.

Deviations from the protocol should not occur. If deviations occur, the Investigator must inform the Study Monitor, and the implications of the deviations must be reviewed and discussed. Any deviation must be documented, stating the reason and date, the action taken, and the impact for the subject and/ or the study. The documentation must be kept in Investigator's Trial Master File and the Sponsor's Trial Master File.

12 ETHICS

The study will be conducted in accordance with the Declaration of Helsinki and the International Conference on Harmonisation GCP guidelines.

12.1 Informed Consent Form for Study Subjects

In obtaining and documenting informed consent, the Investigator must comply with the applicable regulatory requirements and adhere to the ICH GCP guideline and the requirements in the Declaration of Helsinki.

Prior to any study related activity, the Investigator must give the subject and the subject’s parents or guardians oral and written information about the study in a form that they can read and understand. Sufficient time must be given to the subject and their family to discuss the study, away from the investigative site, and to decide whether participation is appropriate.

A voluntarily signed and dated Permission Form will be obtained from the subject’s parents or guardians prior to any study related activity. The Permission Form must be signed by both of the subject’s parents unless one parent is deceased, unknown, incompetent, not reasonably available or where only one parent has legal responsibility for care and custody of the child. The written Permission Form must also be signed and dated by the person who conducted the informed consent procedure.

In addition to the Permission Form, an Assent Form should be signed by the subject, where appropriate. In general, subjects aged 11 to 14 years should read and sign the Assent Form. This form will contain all pertinent information about the trial in age appropriate language. Subjects aged 5 to 10 years should read and sign the Assent Form if they are able and where it is deemed, by the site staff, to be appropriate.

Regardless of the subject’s ability to read or sign Assent Forms, their wishes should be fully considered and if it is at all possible that they do not wish to take part, they should be given the opportunity to express that wish and be excluded from the study. Consideration should be given to the possibility that a child who declines to take part on one occasion, may change his/her mind at a later date and subsequently be a willing participant.

If information becomes available that may be relevant to the subject's willingness to continue participating in the study, the Investigator must inform the subject and their parents in a timely manner, and revised written Permission and Assent forms must be obtained.

12.2 Independent Ethics Committee

Before commencement of the study the following will be submitted to the IEC; the protocol and any amendments, Subject/Parent Information and Permission/Assent Forms, any other written information to be provided to the subject, subject recruitment procedures, if any, Investigator's Brochure (IB), information about payments and compensation available to subjects, if not mentioned in the subject information, the Investigator's current CV and other documents as required. The submission letter should clearly identify (by including version number and date of the document) which documents have been submitted to the IEC. Written approval must be obtained for the IEC prior to commencement of the study.

During the study, the Investigator must promptly report the following to the IEC; updates to IB, unexpected SAEs where a causal relationship cannot be ruled out, amendments to the protocol, notes of administrative changes, deviations to the protocol implemented to eliminate immediate hazards to the study subjects, new information that may affect adversely the safety of the subjects or the conduct of the study, annually written summaries of the study status, and other documents as required by the IEC.

Amendments must not be implemented before approval, unless necessary to eliminate hazards to the subjects.

The Investigator must maintain an accurate and complete record of all submissions made to the IEC. The records should be filed in the Investigator's Trial Master File, and copies must be sent to Emergent Product Development UK Ltd.

12.3 Regulatory Authorities

Regulatory Authorities will receive the protocol, amendments, reports on SAEs, and the Integrated Clinical Trial Report according to national regulations.

13 DATA HANDLING AND RECORD KEEPING

All data entry will be computerised without using subject names.

13.1 Data Entry

Second entry verification will be performed with primary and secondary entry being carried out by separate operators. The two entries will be compared and any discrepancies resolved by reference to the CRF.

Data entry guidelines will be used to assist in the data entry process.

13.2 Data Coding

Adverse events will be coded using MedDRA (version 8.1 or later).

13.3 Audit Trail

SAS datasets will be created and a full audit trail kept to track creations, edits and deletions. Any changes to the data will be tracked per subject.

13.4 Data Validation and Checks

Validation checks will be written in SAS to check the consistency and accuracy of the values. Consistency checks will also be performed as required.

14 FINANCE AND INSURANCE

All financial agreements for the conduct of the study are detailed in contracts between Emergent Product Development UK Ltd (formerly Microscience Ltd) and the Investigator. Details of financial compensation for services rendered will be recorded. The Investigator may reimburse reasonable expenses, incurred by the subjects or their parents, when receipts or a mileage claim supports the expenses.

The Investigator must provide Emergent Product Development UK Ltd with sufficient and accurate financial disclosure information for Emergent Product Development UK Ltd to meet their regulatory disclosure requirements (21CFR54). The clinical Investigator will update this information if any relevant changes occur during the investigation and for one year following completion of the study.

The study site will maintain adequate public liability insurance cover throughout the term of the study and, on request, will provide satisfactory evidence of its adequate insurance cover to Emergent Product Development UK Ltd.

Emergent Product Development UK Ltd will maintain adequate clinical trials liability insurance coverage throughout the term of the study and, on request, will provide satisfactory documentary evidence of its adequate insurance cover to the study site.

15 REPORTS AND PUBLICATIONS

Emergent Product Development UK Ltd recognises that Oxford University and the site Principle Investigator have a responsibility under the Research Governance Framework for Health and Social Care to ensure that results of scientific interest arising from the clinical trial are appropriately published and disseminated. Emergent Product Development UK Ltd agrees that employees of Oxford University will be permitted to present at symposia, national or regional professional meetings and to publish in journals, theses or dissertation or otherwise, of their own choosing, methods and results of the clinical trial, subject to the publication policy described in this protocol.

Upon completion of the clinical trial and prior to publication of multi-centre data, or when the clinical trial data are adequate (in Emergent Product Development UK Ltd's reasonable judgement), Oxford University may prepare the data deriving from the clinical trial for publication. Such data will be submitted to Emergent Product Development UK Ltd for review and comment prior to publication. In order to ensure that Emergent Product Development UK Ltd will be able to make comments and suggestions where pertinent, material for public dissemination will be submitted to Emergent Product Development UK Ltd for review at least sixty (60) days prior to submission for publication, public dissemination or review by publication committee.

Oxford University agrees that it will give fair consideration to all reasonable comments made by Emergent Product Development UK Ltd in relation to a proposed publication by Oxford University but Emergent Product Development UK Ltd recognises that there will be no obligation upon Oxford University to modify the publication.

During the period for review of a proposed publication, referred to above, Emergent Product Development UK Ltd will be entitled to make a reasoned request to Oxford University that publication be delayed for a period of up to six (6) months from the date of its first submission to

Emergent Product Development UK Ltd in order to enable Emergent Product Development UK Ltd to take steps to protect its proprietary information and Oxford University will not unreasonably withhold its consent to such a request.

16 ARCHIVING

The investigator will retain all documents associated with the study for at least 5 years. Medical files of trial subjects shall be retained in accordance with national legislation and in accordance with the maximum period of time permitted by the hospital or institution. Study documents shall be retained for a longer period if required by the applicable regulatory authority or by agreement with the sponsor. If the investigator responsible for the study should move or retire during this period he will nominate someone in writing to be responsible for record keeping

17 REFERENCES

Acharya IL, Lowe CU, Thapa R, Gurubacharya VL, Shrestha MB, Bact D, *et al*. Prevention of typhoid fever in Nepal with the Vi capsular polysaccharide of *Salmonella typhi*. A preliminary report. N Engl J Med 1987; 317: 1101-4.

Chatfield S, Roberts M, Londono P, Cropley I, Douce G, Dougan G. The development of oral vaccines based on live attenuated *Salmonella* strains. FEMS Immunol Med Microbiol 1993; 7: 1-7.

Chuttani CS, Prakash K, Vergese A, Gupta P, Chawla RK, Grover V, *et al*. Ineffectiveness of an oral killed typhoid vaccine in a field trial. Bull World Health Organ 1973; 48: 754-5.

Collins, FM. Vaccines and cell-mediated immunity. Bacteriol Rev 1974; 38: 371-402.

Dupont HL, Hornick RB, Snyder MJ, Dawkins AT, Heiner GG, Woodward TE. Studies of immunity in typhoid fever. Protection induced by killed oral antigens or by primary infection. Bull World Health Organ 1971; 44: 667-72.

Farrington CP, Manning G. Test statistics and sample size formulae for comparative binomial trials with null hypothesis of non-zero risk difference for non-unity relative risk. Statistics in Medicine, 1990; 9:1447-1554

Ferreccio C, Levine MM, Rodriguez H, Contreras R. Comparative efficacy of two, three, or four doses of Ty21a live oral typhoid vaccine in enteric-coated capsules: a field trial in an endemic area. J Infect Dis 1989; 159: 766-9.

Germanier R, Furer E. Isolation and characterization of gal E mutant Ty21a of *Salmonella typhi*: A candidate strain for a live oral typhoid vaccine. J Infect Dis 1975; 131: 553-8.

Gonzalez C, Hone D, Noriega FR, Tacket CO, Davies JR, Losonsky G, *et al*. *Salmonella* typhi vaccine strain CVD 908 expressing the circumsporozoite protein of *Plasmodium falciparum*: strain construction and safety and immunogenicity in humans. J Infec Dis 1994; 169: 927-31.

Hindle Z, Chatfield SN, Phillimore J, Bentley M, Johnson J, Cosgrove CA *et al*. Characterization of *Salmonella enterica* derivatives harboring defined *aro*C and *Salmonella* pathogenicity island 2 type III secretion system (*ssa*V) mutations by immunization of healthy volunteers. Infect Immun 2002; 70: 3457-67.

Hormaeche CE, Joysey HS, Desilva L, Izhar M, Stocker BA. Immunity induced by live attenuated *Salmonella* vaccines. Res Microbiol 1990; 141: 757-64.

Klugman KP, Gilbertson IT, Koornhof HJ, Robbins JB, Schneerson R, Schulz D, *et al*. Protective activity of Vi capsular polysaccharide vaccine against typhoid fever. Lancet 1987; Nov 21; 1165-9.

Klugman KP, Koornhof HJ, Robbins JB, Le Cam NN. Immunogenicity, efficacy and serological correlate of protection of *Salmonella typhi* Vi capsular polysaccharide vaccine three years after immunization. Vaccine 1996; 14: 435-8.

Kossaczka Z, Lin FY, Ho VA, Thuy NT, Van Bay P, Thanh TC *et al*. Safety and immunogenicity of Vi conjugate vaccines for typhoid fever in adults, teenagers, and 2- to 4-year-old children in Vietnam. Infect Immun. 1999 Nov; 67(11): 5806-10.

Levine MM. Typhoid fever vaccines. In SA Plotkin and EA Mortimer, eds. Saunders, Philadelphia, Pennsylvania, USA, Vaccines 1994; 597-633.

Levine MM, Sztein MB. Human mucosal vaccines for *Salmonella typhi* infections. In Kiyono H, Ogra PL and McGhee JR eds; Chapter 15; Academic Press, San Diego, USA, 1996

Lin FY, Ho VA, Khiem HB, Trach DD, Bay PV, Thanh TC *et al*. The efficacy of a Salmonella typhi Vi conjugate vaccine in two-to-five-year-old children. N Engl J Med. 2001; 344: 1263-9.

Lin FY, Vo AH, Phan VB, Nguyen TT, Bryla D, Tran CT, Ha BK, Dang DT, Robbins JB. The epidemiology of typhoid fever in the dong thap province, Mekong delta region of Vietnam. Am. J. Trop. Med. Hyg, 2000; 62(5), 644–8

Lowe DC, Savidge TC, Pickard D, Eckmann L, Kagnoff MF, Dougan G, *et al.* Characterization of candidate live oral *Salmonella typhi* vaccine strains harboring defined mutations in aroA, aroC, and *htrA*. Infect Immun 1999; 67: 700-7.

McGhee JR, Mestecky J, Dertzbaugh MT, Eldridge JH, Hirasawa M, Kiyono H. The mucosal immune system: from fundamental concepts to vaccine development. Vaccine 1992; 10: 75-88.

Nguyen TA, Ha Ba K, Nguyen TD. Typhoid fever in South Vietnam, 1990-1993: Bull Soc Pathol Exot. 1993;86(5 Pt 2):476-8

Ochman H, Soncini FC, Solomon F, Groisman EA. Identification of a pathogenicity island required for *Salmonella* survival in host cells. Proc Natl Acad Sci 1996; 93: 7800-4.

Rao MR, Blackwelder WC, Troendle JF, Naficy AB, Clemens JD. Sample size determination for phase II studies of new vaccines. Vaccine 2002; 20 3364-3369.

Shea JE, Beuzon CR, Gleeson C, Mundy R, Holden DW. Influence of the Salmonella typhimurium pathogenicity island 2 type III secretion system on bacterial growth in the mouse. Infect Immun 1999; 67: 213-9.

Sztein MB, Wasserman SS, Tacket CO, Edelman R, Hine D, Lidberg AA, *et al*. Cytokine production patterns and lymphoproliferative responses in volunteers orally immunized with attenuated vaccine strains of *Salmonella typhi*. J Infect Dis 1994; 170: 1508-17.
